# Evolution of Acquired Perfumes and Endogenous Lipid Secretions in Orchid Bees

**DOI:** 10.1007/s10886-024-01514-w

**Published:** 2024-07-03

**Authors:** Thomas Eltz, Tobias Mende, Santiago R. Ramírez

**Affiliations:** 1 Department of Animal Ecology, Evolution and Biodiversity, Ruhr-Universität Bochum, 44801 Bochum, NRW Germany; 2 Department of Evolution and Ecology and Center for Population Biology, University of California, Davis, CA 95616 USA

**Keywords:** Sexual communication, Sex pheromones, Chemical evolution, Volatile collection, Enfleurage, Euglossini

## Abstract

Male orchid bees are unique in the animal kingdom for making perfumes that function as sex pheromone. Males collect volatile chemicals from the environment in the neotropical forests, including floral and non-floral sources, creating complex but species-specific blends. Male orchid bees exhibit several adaptations to facilitate perfume collection and storage. When collecting volatile compounds, males apply lipid substances that they secrete from cephalic labial glands onto the fragrant substrate. These lipids help dissolve and retain the volatiles, similar to the process of ‘enfleurage’ in the traditional perfume industry. We investigated how the chemical composition of acquired perfume and labial gland secretions varied across the phylogeny of orchid bees, including 65 species in five genera from Central and South America. Perfumes showed rapid evolution as revealed by low overall phylogenetic signal, in agreement with the idea that perfume compounds diverge rapidly and substantially among closely related species due to their role in species recognition. A possible exception were perfumes in the genus *Eulaema*, clustering closely in chemospace, partly mediated by high proportions of carvone and *trans*-carvone oxide. Labial gland secretions, in contrast, showed a strong phylogenetic signal at the genus level, with secretions of *Eufriesea* and *Exaerete* dominated by fatty acids and *Eulaema* dominated by saturated acetates of chain lengths 12 to 16 C-atoms. Secretions of the majority of *Euglossa* were heavily dominated by one unsaturated long chain diacetate, (9*Z*)-Eicosen-1,20-diyldiacetate. However, we also identified few highly divergent species of *Euglossa* in four subclades (11 species) that appear to have secondarily replaced the diacetate with other compounds. In comparison with environment-derived perfumes, the evolution of labial gland secretion is much slower, likely constrained by the underlying biochemical pathways, but perhaps influenced by perfume-solvent chemical interactions.

**Supplementary Information:**

The online version contains supplementary material available at 10.1007/s10886-024-01514-w.

## Introduction

Chemical signals are widespread in sexual communication systems across the tree of life, especially in insects and other arthropods. A traditional view is that semiochemicals involved in mate recognition are conserved in structure and function through stabilizing selection, maintaining reproductive isolation among existing lineages. However, some studies have suggested that changes in biosynthetic pathways can trigger substantial changes in the signal (Roelofs et al. 2002), which in turn can lead to bursts of diversification and the evolution of reproductive isolation (Cama et al. 2022). Furthermore, selection against costly hybrid mating (reinforcement) can drive the rapid divergence of chemical signals when closely related taxa occur in sympatry (Weber et al. 2016). In the present study we traced the evolution and diversification of two rather distinct sets of chemicals involved in sexual signaling in neotropical orchid bees (Apinae, Euglossini): the “perfumes”, blends of volatiles that male bees collect from diverse sources in their habitat, and the self-produced labial gland lipids which males use to dissolve and store the exogenous volatiles.

Orchid bees are a group of conspicuously colored, medium-sized to large bees comprising + 230 species in five genera from Central and South America (Ramirez 2009). They are best known for the specific pollination of approximately 800 + species of neotropical orchids by scent-seeking males, a mutualistic interaction that has gained much attention from pollination biologists (Dressler 1968; Ramírez et al. 2011; Williams 1982). However, sources of volatiles for male orchid bees also include flowers of many non-orchid plants as well as non-floral substrates, *e.g.* decaying wood and vegetation, sap from tree wounds or feces (Whitten et al. 1993). Male orchid bees take up volatiles using specialized brushes of hairs on their fore tarsi and store them in hair-filled cavities on their hind legs (tibiae) (Vogel 1966). The uptake is facilitated by the application of lipid secretions from the cephalic labial glands (LG from here on) onto the fragrant substrate (Whitten et al. 1989). Cephalic labial glands of male euglossines are large and shaped like grapes with two lobes in each horizontal half of the head, filling much of the space around the brain (Eltz et al. 2007). Previous studies have shown that the LG secretion is composed of mixtures of aliphatic compounds including saturated and unsaturated straight chain hydrocarbons, alcohols, acetates and esters (Whitten et al. 1989, 1993; Williams and Whitten 1983). It has been suggested that LG lipids serve to dissolve and retain the volatile compounds and thus increase efficiency of volatile uptake, much in the same way as does the application of animal fat in “enfleurage”, a method of floral scent extraction in the traditional perfume industry (Whitten et al. 1989).

Chemical analyses of the tibial contents show that both LG lipids and volatiles are incorporated and mixed in the tibial pouches of odor-collecting male euglossines (Whitten et al. 1989), with the proportion of volatiles to lipids varying substantially between species and with time since the last access to volatiles (Eltz 1997). Earlier ideas that either perfumes or lipids (or both) are chemically altered within the pouch have not been supported by data, and it appears that tibial containers are primarily volatile storage devices (Eltz et al. 2019; Whitten et al. 1989). However, experiments with synthetic Deuterium-labeled tracer compounds have shown that, over hours to days after volatile uptake, LG lipids are selectively relocated from the containers and moved back to the labial glands where they are used in consecutive bouts of volatile collection (Eltz et al. 2007). This relocation is presumed to occur through the hemolymph with the help of lipophorin carrier proteins (Whitten et al 1989), which are also involved in transporting cuticular hydrocarbons (Chino and Downer 1982).

While lipids are relocated from the hind-legs to the labial glands, continued volatile collection leads to the accumulation of a complex blend of exogenous compounds in the hind tibiae referred to as perfume. Although there is also substantial variability among individuals, perfume mixtures have been found to be species-specific in chemical composition, even across different localities, regions and seasons (Brand et al. 2020; Darragh et al. 2023; Eltz et al. 1999; Ramírez et al. 2010a; Zimmermann et al. 2009b). In the largest genus, *Euglossa*, phylogeny-based studies indicate that perfume composition has diverged more rapidly than expected by neutral evolution among closely related species (Zimmermann et al. 2009a), and that divergence is particularly pronounced when species occur in sympatry (Weber et al. 2016). The combined evidence suggests that perfumes are selected to function in mate choice and species recognition, an idea that is also supported by behavioral observations and experiments. Males expose perfumes during a characteristic display behavior in small, non-resource based mating territories (Eltz et al. 2005), and they do so positioned in a way ideal for signaling to conspecifics approaching from downwind (Pokorny et al. 2017). Mating events are rarely observed in nature, but recent experiments in large flight cages have finally provided evidence that male perfume signaling is necessary for attracting females in the context of mating (Henske et al. 2023).

While previous studies have investigated the evolution of perfume disparity across a large number of individuals and species in the genus *Euglossa* (Weber et al. 2016), a comparison of perfumes across euglossine genera has not been conducted. In the present study we fill this gap, analyzing species-level phylogenetic and chemical data from all five euglossine genera: *Eufriesea*, *Exaerete*, *Aglae*, *Eulaema* and *Euglossa*. In addition, we present the first detailed comparison of the chemical composition of LG lipids across genera. We ask whether lipids and perfumes exhibit different rates of evolution across species and genera, and whether lipid and perfumes coevolve to optimize volatile uptake.

## Methods and Materials

### Samples

We lured male orchid bees to chemical baits at a range of neotropical localities and analyzed labial gland (LG) and hind tibial perfume compounds (P) (see below for details). Localities and sampling procedures were identical to those used in a previous study on perfume evolution in the genus *Euglossa* see Weber et al. (2016), including a map of sampling localities. In contrast to Weber et al. (2016), who also tested species specificity within *Euglossa*, the present study is restricted to species level variation, i.e. we use LG and perfume compositional data averaged across individuals of a given species. The species and data of *Euglossa* are a subset of those in Weber et al. (2016), see below, including the species that had at least three individual samples for LG or P (49 species in LG, 48 in P). In addition, we newly analyzed samples from the four other euglossine genera: *Exaerete* (two species in LG and P), *Eufriesea* (5 species in LG and 7 in P), *Aglae* (1 species in both sets) and *Eulaema* (five species in both sets). Overall, our data included 65 species of Euglossini, 62 in the LG and 63 in P data sets. The number of individual samples per species varied between 3 to 40 in LG and 3 to 28 in P.

For the genus *Euglossa* the data sets were modified with respect to Weber et al. (2016) in the following way: Two individuals incorrectly identified as *Euglossa viridifrons* by Weber et al. (2016) were now included as *Euglossa allosticta*. Also, we newly collected 7 males of *Euglossa piliventris* in 2021 and included the species in LG. Based on its LG profile we also re-included the closely related *Euglossa lugubris* in LG. This individual had been excluded in Weber et al. (2016) because its unusual LG composition had been considered an artifact at the time. It is the only exception to the rule that only species with at least 3 individual samples were included.

A list of all individual samples (685 in P, 663 in LG) used for averaging species profiles along with sampling localities is given in Supplementary Information 1.

### Sampling and Sample Preparation

Pure synthetic chemicals were used to bait male orchid bees: 1,8-cineole, methyl salicylate, p-dimethoxybenzene, methyl cinnamate, skatol, p-cresol, vanillin, β-ionone, benzyl acetate, ipsdienol and eugenol. These are chemicals that are well known to be attractive to and to be collected by males of variable numbers of species of orchid bees in pure form (Roubik and Hanson 2004). The chemicals were exposed on paper tissue covered with screen mesh (usually in metal tea sifters) to prevent male bees from directly accessing the chemicals. Males were captured with hand nets and killed by freezing. Right hind legs, including the right perfume container, were removed and extracted with 500 μl of hexane in 2 ml screw-cap autosampler vials. To obtain samples of LG lipids, a large part of the head around the right eye was cut off and extracted in 500 μl of hexane in a separate vial. Previous dissection had shown that cephalic labial glands fill much of the space around the brain and eyes in male euglossine bees (Eltz et al. 2007), and cutting off the head capsule around an eye provided a simple way of obtaining labial gland extracts for large numbers of bees. It also avoided sampling secretions of the mandibular gland located at the base of the mandibles. Voucher specimens of all samples were pinned and deposited in the reference collection of Thomas Eltz.

### Chemical Analysis

Gas Chromatography/Mass Spectrometry (GC/MS) was conducted at the Department of Animal Ecology, Evolution and Biodiversity, Ruhr-University Bochum, using a HP 5890 II GC coupled with a 5972 Mass Selective Detector (Agilent Technologies, Santa Clara, CA, USA). The GC was fitted with a 30 m long, 0.25 mm ID, non-polar Agilent HP-5MS capillary column Sample aliquots of 1 μl were injected splitless by using an Agilent 7673B automatic injector. The oven temperature was programmed from 60 to 300 °C at 10 °C/min, using helium as carrier gas.

We used MSD Chemstation (Build 75, v. 01.00, Agilent Technologies) to call chromatogram peaks and to save their corresponding spectra in a user mass-spectral library that allowed us to cross-reference spectra peaks across all chromatograms. The library was updated as new compounds were found. We characterized individual compounds by comparing spectra and retention indices against published libraries (Adams 2001; Ausloos et al. 1992) or those of authentic standards. Peak areas (integrated ion currents) were standardized across compounds to provide % contribution of a compound to an individual perfume or labial gland secretion profile.

Male hind leg extracts contain both exogenous volatiles of variable chemical affiliation plus endogenous aliphatic compounds. These aliphatic compounds include large quantities of labial glad lipids as well as traces of typical cuticular hydrocarbons (Pokorny et al. 2014, 2015). These aliphatic compounds in hind leg extracts were considered as endogenous and not part of the perfume profile (P). For downstream analysis we therefore used two chemical datasets: Aliphatic labial gland profiles (LG) which included all compounds found in the labial gland extracts, and perfume profiles (P) which included compounds found in the hind leg extracts minus the compounds also found in LG extracts and minus typical long chain cuticular hydrocarbons.

### Data Analysis

We averaged individual relative peak areas for each species with at least three samples, thus compiling species-level standardized data of LG and P profiles. These data were used for downstream analysis and placed in a phylogenetic context using a fossil time-calibrated molecular phylogeny for Euglossini (Ramírez et al. 2011).

To assess chemical disparity among species we square root transformed LG and P profiles and calculated the *Bray–Curtis index* of dissimilarity between species. Pair-wise dissimilarities were reduced in dimensionality using the non-metric *Multidimensional Scaling (MDS)* functions nmds and nmds.min in the package ‘ecodist’ (Goslee and Urban 2007). We visualized patterns of evolutionary divergence and phylogenetic structure with phylo-chemospace plots, where the two-dimensional chemical trait space relates to lines according to the phylogenetic relationships of species. For this we used the *phylo-morphospace function* in the phytools package (Revell 2012). To further explore how single chemical compounds contribute to chemical disparity of species and genera we overlaid *MDS* plots with “bubbles” representing averaged relative peak areas of selected chemicals using the function ggplot in ggplot2 (Wickham 2016).

To compare the evolutionary dynamics of LG and P profiles, we plotted pairwise chemical disparity of species as a function of phylogenetic distance taken from (Ramírez et al. 2011).

We tested for effects of clade/genus affiliation on differences in chemical composition across species using *ANOSIM permutation tests* in the software PRIMER (Clarke and Gorley 2001; Clarke and Warwick 2001). The same software (SIMPER algorithm) was used to calculate the contribution of individual compounds to the overall chemical similarity within groups of species.

Finally, we searched for correlations between LG and P profiles using the RELATE function (Mantel test) in PRIMER v5 (Clarke and Gorley 2001; Clarke and Warwick 2001) to evaluate the possibility of coevolution between the two sets of chemicals.

## Results

**Fig. 1:**
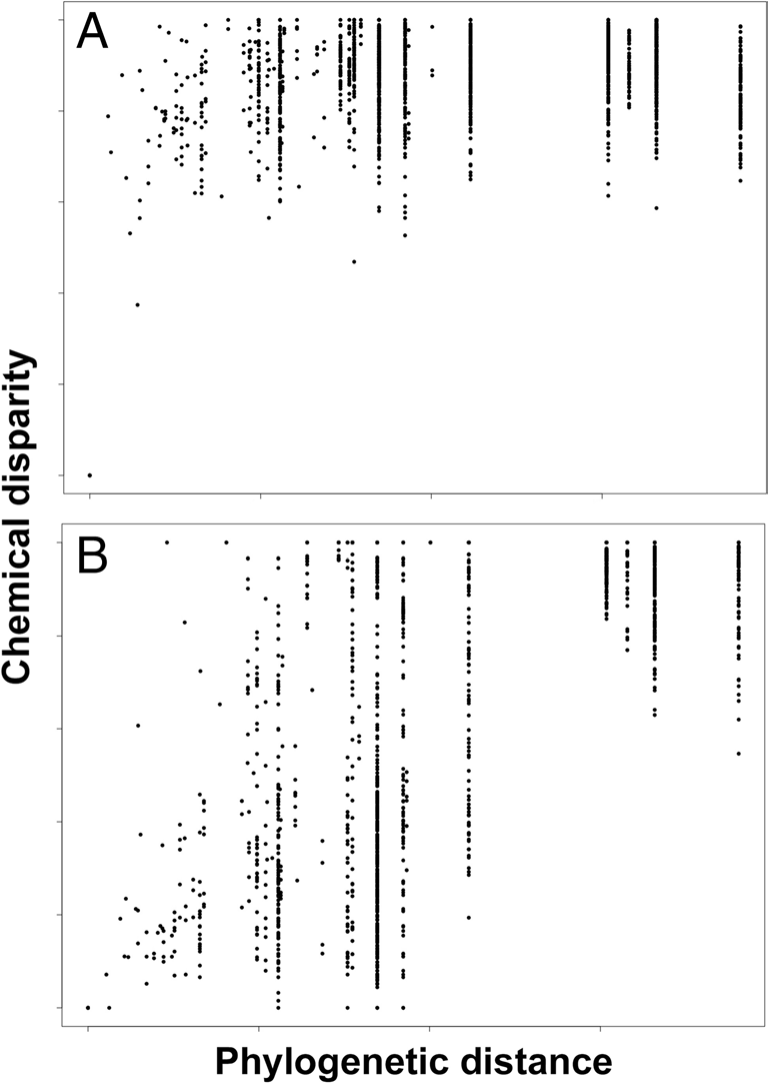
Relationship between standardized phylogenetic distance and standardized chemical disparity of (**A**) male perfumes and (**B**) labial gland secretions between species orchid bees. Each dot represents one species pair from a total of 63 (in A) and 62 (in B). Note strong phylogenetic signal in the disparity of labial gland secretions

**Fig. 2:**
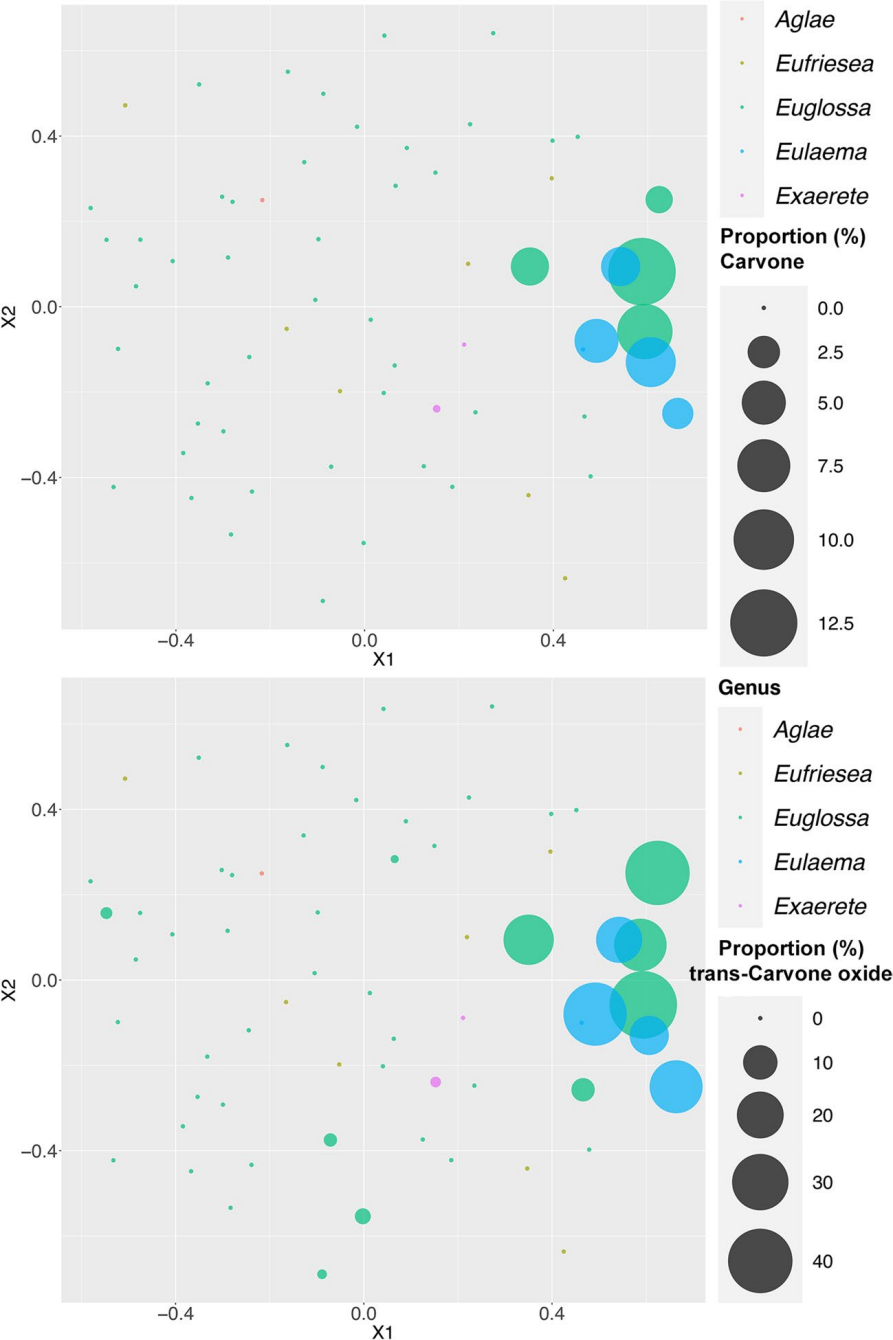
Two-dimensional chemospaces of male hind tibial perfumes of 63 species of orchid bees (Euglossini), superimposed with relative proportion of carvone (top) and *trans*-carvone oxide (bottom) proportions in profiles. Distances between individual dots (species) represent dissimilarity (Bray–Curtis) of chemical profiles among species reduced in dimensionality with n-MDS. Euglossine genera are color coded according to panel

Perfumes (P, 63 species, 599 compounds, Supplementary Information 2) and labial gland lipids (LG, 62 species, 154 compounds, Supplementary Information 3) showed very different patterns of evolutionary divergence and phylogenetic structure. Chemical disparity of perfumes (P) was found throughout all levels of the phylogenetic tree (Supplementary Information 4), with rapid divergence between closely related species resulting in a low overall pylogenetic signal (Fig. 1A). Rapid divergence of perfumes was particularly evident within the most speciose genus, *Euglossa*, exemplified by the highly divergent sibling species pairs *Euglossa purpurea*/*Eug. hansoni* and *Eug. crassipunctata*/*Eug. sapphirina*. As a result, little subgenus phylogentic structure in perfume composition was observed within *Euglossa*. The six species included in the subgenus *Glossura* (*Euglossa chalybeata*, *Eug. orellana*, *Eug. occidentalis*, *Eug. flammea*, *Eug. ignita* and *Eug. imperialis*) appeared to cluster in two-dimensional MDS space, but such an effect was not supported by ANOSIM (*Glossura* vs. the remaining *Euglossa*: R = 0.09; N.S.). Due to lower species number/taxon sampling, our data set included fewer sibling pairs in the other genera, but there was at least one example of pronounced perfume divergence between two closely related species: *Eufriesea concava* and *Euf. mussitans* perfumes were as distinct from each other as those of more distantly related *Eufriesea*. All five species of *Eulaema* clustered closely in two-dimensional perfume space, as did the two included species of *Exaerete* (Supplementary Information 4). Overall, there was a weak but significant effect of the factor ‘genus’ on perfume (P) disparity (ANOSIM R = 0.19, *p* < 0.05). The high within-group similarity of *Eulaema* perfumes was partly mediated by a shared dominance of the structurally related compounds carvone (2.3 to 6.6% peak area) and *trans*-carvone oxide (13.5 to 38.3% peak area), together contributing 38.3% to overall within-group perfume similarity. However, the same compounds were also present in similar relative abundances in some species of *Euglossa* (Fig. 2). Our nomenclature of carvone oxides follows Brandt et al. (2019), with the peak of trans-carvone oxide eluting briefly before the peak of cis-carvone oxide on DB-5. A differentiation of enantiomers within either of the two diastereomers was not possible with our analytical method.

**Fig. 3:**
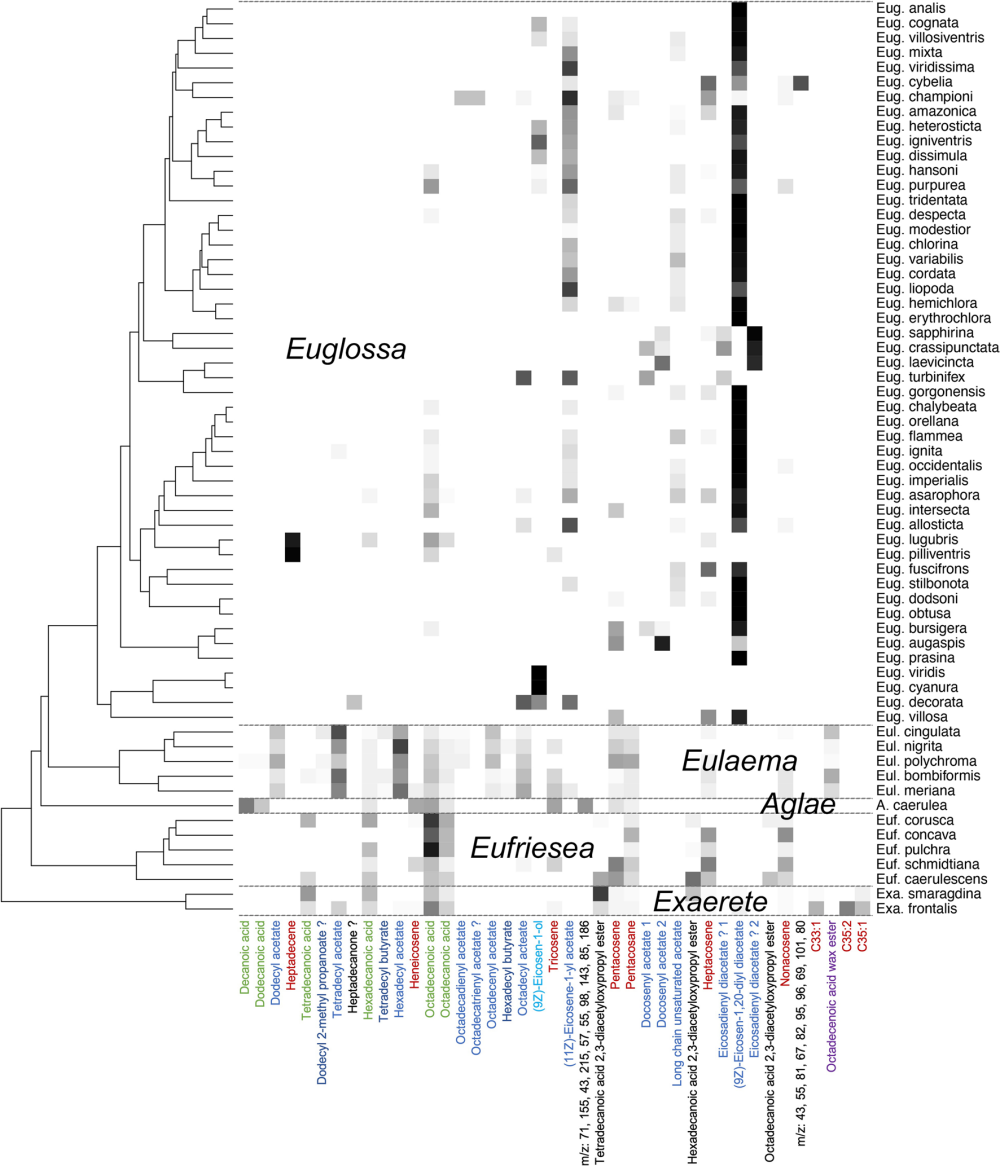
Relative abundance of the 41 most abundant compounds in labial gland (LG) extracts across five genera and 62 species of orchid bees (Euglossini). Phylogeny modified from (Ramírez et al. 2011). Compound names are colored by substance class: Fatty acids (green), alkanes and alkenes (red), long chain alcohol (bright blue), long chain monoacetates and diacetates (dark blue), other long chain esters (even darker blue), and triaglycerols and unidentified compounds (black). The most abundant ten EI ion masses are given for unknown structures. Grey scale represents square root transformed proportions from 0 (white) to 100% (black) of averaged standardized peak area

Labial gland lipids (LG) showed a completely different pattern of divergence, exhibiting a strong phylogentic signal (Fig. 1B) with pronounced between-genus differences in composition (Supplementary Information 4; ANOSIM R = 0.57, *p* < 0.001). There were differences in the overall diversity of labial gland compounds among genera, with the highest number of detected compounds in *Exaerete* (40, 57) and *Eulaema* (45 ± 9) species, intermediate numbers in *Eufriesea* (20 ± 10) and *Aglae* (16) and low diversity in Euglossa (4 ± 3) species. This variability reflects body (and probably gland) size, and may partly be caused by more peaks/compounds jump over the GC/MS detection threshold in more concentrated extracts. It should be noted that the compound set included straight chain hydrocarbons, alkanes and alkenes, some of which may have been derived from the cuticle around the eye (cuticular hydrocarbons). We included straight chain hydrocarbons in LG because in some species certain compounds were extracted in large quantities, suggesting they were derived from the labial glands and integral parts of the secretion. Figure 3 visualizes relative abundances of the 41 most abundant LG compounds across the phylogeny of Euglossini.

Extracts of *Exaerete* spp. and *Eufriesea* spp., the early branching lineages of Euglossini in our phylogeny (Ramírez et al. 2010b), were dominated by saturated and unsaturated fatty acids, particularly (9*Z*)-Octadec-9-enoic acid (oleic acid). These lineages also contained certain long chain hydrocarbons (alkanes and alkenes; Fig. 3). *Exaerete* and some *Eufriesea* spp. in addition contained variable quantities of three homologous triacylglycerols (straight chain C14-, C16-, C18 saturated fatty acid 2,3-diacetyloxypropyl esters).

*Aglae caerulea* from the monotypic genus *Aglae* also mostly contained fatty acids, notably the ones with the shortest chain lengths in the set (decanoic and dodecanoic acid), along with alkanes and alkenes. It also had intermediate quantities of an unidentified compound.

Extracts of the two most derived genera, *Eulaema* and *Euglossa*, were dominated by acetates (Fig. 3). In the case of *Eulaema* these were strait chain saturated acetates of chain lengths of 12 to 16 C-atoms. In contrast, LG secretions of the large majority of *Euglossa* were heavily dominated by a single unsaturated long chain diacetate, (9*Z*)-Eicosen-1,20-diyldiacetate, which has previously been found in a range of *Euglossa* species (Eltz et al. 2003, 2007, 2019; Whitten et al. 1993). A minority of *Euglossa* clades did not show a predominance of (9*Z*)-Eicosen-1,20-diyldiacetate: (1) All three members of the early-branching *viridis*-species group produce large amounts of (9*Z*)-Eicosen-1-ol or long chain monoacetates. (2) *Euglossa piliventris* and its near-cryptic sibling from the Western Amazon, *Eug. lugubris*, are highly unusual for having heptadecene as their major LG lipid. (3) A closely related group of species from the subgenus *Glossurella*, including *Euglossa crassipunctata* and *Eug. sapphirina*, appear to have lost (9*Z*)-Eicosen-1,20-diyldiacetate in favor of a doubly unsaturated homologue. Finally, (4) *Euglossa championi* and *Eug. cybelia* retain only reduced amounts of (9*Z*)-Eicosen-1,20-diyldiacetate in favor of other lipids.

Although the dynamics of divergence was quite different between perfume and LG profiles (see Supplementary Information 4 and Fig. 1), we identified a positive correlation between the underlying similarity matrices of the two sets of chemicals (Mantel test: R_s_ = 0.28; *p* < 0.01).

## Discussion

In our analysis we found very distinct patterns of disparity in male perfume (P) and labial gland lipid (LG) composition across the euglossine phylogeny. With regard to the environment-derived perfumes our results broadly confirm previous findings of rapid divergence across the genus *Euglossa*, even among closely related species pairs (Weber et al. 2016; Zimmermann et al. 2009a). Although based on lower species sampling, a similar pattern was found in the genus *Eufriesea*. In contrast, the included species of *Eulaema* clustered rather closely in 2D perfume space based on similar proportions of some major perfume compounds, notably the structurally related carvone and *trans*-carvone oxide. Preferences for carvone-derived compounds could be a commonality inherent to the genus *Eulaema*. While our restricted sample of species allows no strong conclusions, other studies have found carvone oxides in floral scents of *Eulaema*-pollinated orchids and other plants (Brandt et al. 2019; Whitten et al. 1986). These studies demonstrated behavioral attraction to carvone oxides for at least three additional species of *Eulaema* that were not included in our study, including two from the Atlantic Rainforests in Brazil (Brandt et al. 2019). This suggests that the preferences for carvone and carvone oxides is widespread in the genus *Eulaema*. If so, carvone-derived floral scents could represent a chemical sub-syndrome within euglossophilous plants (Brandt et al. 2019), potentially based on a preference of *Eulaema* females for carvone-derived compounds in male perfumes.

While male perfumes of extant orchid bees showed only a weak phylogenetic signal, the opposite was true for labial gland secretions, for which our study presents the first comprehensive phylogenetic analysis. Most species, at least of the more speciose genera, *Euglossa*, *Eulaema* and *Eufriesea*, could be assigned to its genus based on composition of LG extracts alone. Whereas the labial gland secretions of the early-branching genera *Eufriesea*, *Aglae* and *Exaerete* were dominated by aliphatic acids, those of *Eulaema* contained large amounts of long-chain monoacetates, and those of *Euglossa* were heavily dominated by one specific diacetate, (9*Z*)-Eicosen-1,20-diyldiacetate. The broad distribution of that compound throughout the genus *Euglossa* as well as the fact that it is present at the base of the earliest-branching subclade (*Dasystilbe*: *Euglossa villosa*) suggest that this corresponds to a synapomorphy of the genus *Euglossa*. It should be noted that (9*Z*)-Eicosen-1,20-diyldiacetate was occasionally found in trace amounts in individual samples of non-*Euglossa* species. However, these males have most likely acquired the compound by collecting volatiles at sources that were visited by male *Euglossa* at the same time. In such a situation it is conceivable that allochtonous lipids are transferred to the hind leg containers along with autochtonous ones. Since the process of lipid recycling in male euglossines is broadly targeted at long chain aliphatic lipids (Eltz et al. 2007), small amounts of these allochtonous lipids would also be recycled to the labial glands.

It remains to be seen if the differences in labial gland chemistry between genera and species are of functional significance. Male orchid bees use these lipoid substances during the process of volatile uptake. LG lipids are spread over the fragrant surface in large quantities, using pre-tarsal brushes, presumably to dissolve or simply capture the volatiles that would otherwise be difficult to harvest. An intriguing question is whether the uptake of volatiles of certain chemical properties, e.g. of certain polarity, is linked to certain classes of carrier lipids. For example, the predominance of long chain acetates and diacetates in the derived genera *Eulaema* and *Euglossa* is suggestive of functional adaptation, where the oxygen-containing electrophilic acetate group may facilitate dissolving or retaining polar volatiles. In the present study we found that the pairwise similarity of LG profiles between species is positively correlated with the pairwise similarity of perfume profiles. This correlation is in general agreement with a scenario of coevolution between perfume preferences and labial gland chemistry, but could also reflect constrained neutral evolution of both trait sets. Experimental studies that measure the efficiency of volatile extraction or retention are needed to demonstrate a functional link between specific labial gland lipids and certain perfume compounds or compound classes.

## Supplementary Information

Below is the link to the electronic supplementary material.

Supplementary file1 Supplementary Information 1: List of all individual samples included in the analyses with sampling localities. (PDF 222 KB)

Supplementary file2 Supplementary Information 2: Raw data table of standardized perfume composition averaged per species (63 species x 599 compounds). (TXT 102 KB)

Supplementary file3 Supplementary Information 3: Raw data table of standardized labial gland lipid composition averaged per species (62 species x 154 compounds). (TXT 27 KB)

Supplementary file4 Supplementary Information 4 (DOCX 1.03 MB)

## Data Availability

Raw data sets of perfume and cephalic glandular secretions are given as Supplementary Information 2 and 3.
