# Supplementary material for: Evolution of Acquired Perfumes and Endogenous Lipid Secretions in Orchid Bees
Source: J Chem Ecol. 2024 Jul 3;50(9-10):430–8. doi: 10.1007/s10886-024-01514-w (PMC11493807; doi:10.1007/s10886-024-01514-w)
Supplement: Supplementary file 1 — Supplementary file1 Supplementary Information 1: List of all individual samples included in the analyses with sampling localities. (PDF 222 KB) [file 10886_2024_1514_MOESM1_ESM.pdf]

Supplementary information 1: Sampling localities of individual male orchid bees used for averaging species level chemical composition of perfume (P) and labial gland secretion (LG).

Genus	Species	Country	Latitude	Longitude	ID #	P	LG
Aglae	caerulea	Ecuador	-0,79913	-75,51029	CAH-241	x	x
Aglae	caerulea	Guyane	4,78401	-52,43075	1361	x	x
Aglae	caerulea	Guyane	4,78401	-52,43075	1422	x	x
Aglae	caerulea	Guyane	5,09747	-52,69425	1435	x	x
Aglae	caerulea	Guyane	5,09747	-52,69425	1439	x	x
Eufriesea	anisochlora	Panama	9,12851	-79,71529	1222	x	
Eufriesea	anisochlora	Panama	8,85991	-79,87868	1200	x	
Eufriesea	anisochlora	Panama	8,85991	-79,87868	1202	x	
Eufriesea	caerulescens	Mexico	17,07920	-97,86740	681	x	x
Eufriesea	caerulescens	Mexico	17,04520	-96,75491	209	x	x
Eufriesea	caerulescens	Mexico	17,04520	-96,75491	539	x	x
Eufriesea	concava	Costa Rica	8,70342	-83,18755	1608	x	x
Eufriesea	concava	Costa Rica	8,70342	-83,18755	1609	x	x
Eufriesea	concava	Costa Rica	8,70342	-83,18755	1610	x	x
Eufriesea	concava	Costa Rica	8,70342	-83,18755	1611	x	x
Eufriesea	concava	Costa Rica	8,70342	-83,18755	1612	x	x
Eufriesea	concava	Costa Rica	8,70342	-83,18755	1613	x	x
Eufriesea	corusca	Panama	9,15210	-79,84648	283	x	x
Eufriesea	corusca	Panama	9,15210	-79,84648	284	x	x
Eufriesea	corusca	Panama	9,15210	-79,84648	285	x	x
Eufriesea	corusca	Panama	9,15210	-79,84648	296	x	x
Eufriesea	corusca	Panama	9,15210	-79,84648	297	x	x
Eufriesea	corusca	Panama	9,15210	-79,84648	299	x	x
Eufriesea	corusca	Panama	9,12851	-79,71529	1220	x	
Eufriesea	corusca	Panama	8,85991	-79,87868	1201	x	
Eufriesea	mussitans	Guyane	4,54893	-52,49186	1323	x	
Eufriesea	mussitans	Guyane	4,54893	-52,49186	1329	x	
Eufriesea	mussitans	Panama	8,85991	-79,87868	1203	x	
Eufriesea	pulchra	Panama	9,15210	-79,84648	279	x	x
Eufriesea	pulchra	Panama	9,15210	-79,84648	280	x	x
Eufriesea	pulchra	Panama	9,15210	-79,84648	282	x	x
Eufriesea	pulchra	Panama	9,15210	-79,84648	300	x	x
Eufriesea	pulchra	Panama	9,15210	-79,84648	314	x	x
Eufriesea	pulchra	Guyane	4,54893	-52,49186	1299	x	x
Eufriesea	pulchra	Colombia	6,08334	-77,41670	CAH-155	x	x
Eufriesea	pulchra	Colombia	6,08334	-77,41670	CAH-184	x	x
Eufriesea	pulchra	Colombia	6,08334	-77,41670	CAH-185	x	x
Eufriesea	pulchra	Colombia	6,08334	-77,41670	CAH-186	x	x
Eufriesea	pulchra	Colombia	6,08334	-77,41670	CAH-187	x	x
Eufriesea	pulchra	Guyane	4,53861	-52,13694	1291	x	x
Eufriesea	pulchra	Guyane	4,53861	-52,13694	1292	x	x
Eufriesea	pulchra	Panama	9,12851	-79,71529	516	x	
Eufriesea	pulchra	Panama	8,85991	-79,87868	1204	x	

Eufriesea	schmidtiana	Panama	9,15210	-79,84648	287	x	x
Eufriesea	schmidtiana	Panama	8,85991	-79,87868	359	x	
Eufriesea	schmidtiana	Panama	8,85991	-79,87868	360	x	x
Eufriesea	schmidtiana	Panama	8,85991	-79,87868	361	x	x
Eufriesea	schmidtiana	Panama	8,85991	-79,87868	362	x	x
Eufriesea	schmidtiana	Panama	8,85991	-79,87868	363	x	
Euglossa	allosticta	Colombia	6,08334	-77,41670	CAH-199	x	x
Euglossa	allosticta	Colombia	6,08334	-77,41670	CAH-227	x	x
Euglossa	allosticta	Costa Rica	8,70342	-83,18755	467	x	x
Euglossa	allosticta	Costa Rica	8,70342	-83,18755	468	x	x
Euglossa	allosticta	Costa Rica	8,70342	-83,18755	1636	x	x
Euglossa	allosticta	Costa Rica	8,70342	-83,18755	1645		x
Euglossa	allosticta	Costa Rica	8,70342	-83,18755	1666		x
Euglossa	allosticta	Panama	9,55390	-79,65580	1182	x	
Euglossa	allosticta	Panama	8,85991	-79,87868	364	x	
Euglossa	allosticta	Panama	8,85991	-79,87868	1198	x	
Euglossa	allosticta	Costa Rica	10,39896	-84,13506	449	x	x
Euglossa	amazonica	Ecuador	-0,79913	-75,51029	CAH-281		x
Euglossa	amazonica	Colombia	-4,20543	-69,93281	CAH-131		x
Euglossa	amazonica	Colombia	-4,20543	-69,93281	CAH-133		x
Euglossa	amazonica	Colombia	-4,20543	-69,93281	CAH-134		x
Euglossa	amazonica	Guyane	4,54893	-52,49186	1301	x	
Euglossa	amazonica	Guyane	4,78401	-52,43075	1230	x	
Euglossa	amazonica	Guyane	4,78401	-52,43075	1233	x	
Euglossa	amazonica	Guyane	4,78401	-52,43075	1254	x	
Euglossa	amazonica	Guyane	4,78401	-52,43075	1366	x	x
Euglossa	amazonica	Guyane	4,78401	-52,43075	1380	x	
Euglossa	amazonica	Guyane	4,78401	-52,43075	1383	x	x
Euglossa	amazonica	Guyane	4,78401	-52,43075	1386	x	
Euglossa	amazonica	Guyane	4,78401	-52,43075	1390	x	
Euglossa	amazonica	Guyane	4,78401	-52,43075	1578	x	
Euglossa	amazonica	Guyane	5,09747	-52,69425	1514	x	
Euglossa	amazonica	Guyane	5,09747	-52,69425	1515	x	
Euglossa	amazonica	Guyane	5,09747	-52,69425	1520	x	x
Euglossa	amazonica	Guyane	5,09747	-52,69425	1521		x
Euglossa	amazonica	Guyane	5,09747	-52,69425	1527	x	
Euglossa	amazonica	Guyane	4,83111	-52,44389	1354	x	
Euglossa	amazonica	Guyane	5,15626	-52,88575	1404	x	
Euglossa	analis	Guyane	4,78401	-52,43075	1388	x	x
Euglossa	analis	Guyane	4,78401	-52,43075	1542	x	
Euglossa	analis	Guyane	4,30968	-52,13418	1489	x	
Euglossa	analis	Guyane	4,30968	-52,13418	1492	x	x
Euglossa	analis	Guyane	4,30968	-52,13418	1495	x	x
Euglossa	analis	Guyane	5,09747	-52,69425	1476	x	
Euglossa	analis	Guyane	5,09747	-52,69425	1479	x	x
Euglossa	analis	Guyane	5,15626	-52,88575	1405	x	x
Euglossa	analis	Guyane	5,15626	-52,88575	1414		x

Euglossa	asarophora	Costa Rica	9,62626	-82,84993	1671		x
Euglossa	asarophora	Costa Rica	9,62626	-82,84993	1677		x
Euglossa	asarophora	Costa Rica	9,62626	-82,84993	1682		x
Euglossa	asarophora	Colombia	6,08334	-77,41670	CAH-201		x
Euglossa	asarophora	Colombia	6,08334	-77,41670	CAH-202	x	x
Euglossa	asarophora	Colombia	6,08334	-77,41670	CAH-203	x	x
Euglossa	asarophora	Colombia	6,08334	-77,41670	CAH-204	x	x
Euglossa	asarophora	Panama	8,85991	-79,87868	326	x	x
Euglossa	asarophora	Panama	8,85991	-79,87868	327	x	x
Euglossa	asarophora	Panama	8,85991	-79,87868	328	x	x
Euglossa	asarophora	Panama	8,85991	-79,87868	329	x	x
Euglossa	asarophora	Panama	8,85991	-79,87868	330	x	
Euglossa	asarophora	Panama	8,85991	-79,87868	331	x	
Euglossa	asarophora	Panama	8,85991	-79,87868	332	x	
Euglossa	asarophora	Panama	8,85991	-79,87868	333	x	
Euglossa	asarophora	Panama	8,85991	-79,87868	334	x	
Euglossa	asarophora	Costa Rica	10,39896	-84,13506	451	x	
Euglossa	augaspis	Guyane	4,54893	-52,49186	1295	x	x
Euglossa	augaspis	Guyane	4,54893	-52,49186	1300		x
Euglossa	augaspis	Guyane	4,54893	-52,49186	1302		x
Euglossa	augaspis	Guyane	4,54893	-52,49186	1305		x
Euglossa	augaspis	Guyane	4,54893	-52,49186	1307		x
Euglossa	augaspis	Guyane	4,54893	-52,49186	1314	x	x
Euglossa	augaspis	Guyane	4,54893	-52,49186	1316		x
Euglossa	augaspis	Guyane	4,54893	-52,49186	1324		x
Euglossa	augaspis	Guyane	4,78401	-52,43075	1226	x	
Euglossa	augaspis	Guyane	4,78401	-52,43075	1235	x	
Euglossa	augaspis	Guyane	4,78401	-52,43075	1236	x	
Euglossa	augaspis	Guyane	4,78401	-52,43075	1238	x	
Euglossa	augaspis	Guyane	4,78401	-52,43075	1259	x	
Euglossa	augaspis	Guyane	4,78401	-52,43075	1262	x	
Euglossa	augaspis	Guyane	4,78401	-52,43075	1374		x
Euglossa	augaspis	Guyane	4,78401	-52,43075	1576	x	
Euglossa	augaspis	Guyane	5,09747	-52,69425	1461		x
Euglossa	augaspis	Guyane	4,83111	-52,44389	1343	x	x
Euglossa	augaspis	Guyane	4,83111	-52,44389	1347	x	x
Euglossa	augaspis	Guyane	4,83111	-52,44389	1351		x
Euglossa	augaspis	Guyane	4,83111	-52,44389	1353	x	
Euglossa	augaspis	Guyane	4,83111	-52,44389	1356	x	x
Euglossa	augaspis	Guyane	4,83111	-52,44389	1357		x
Euglossa	augaspis	Guyane	5,15626	-52,88575	1402	x	x
Euglossa	augaspis	Guyane	5,15626	-52,88575	1413		x
Euglossa	augaspis	Guyane	5,15626	-52,88575	1420		x
Euglossa	bursigera	Ecuador	-0,79913	-75,51029	CAH-307		x
Euglossa	bursigera	Colombia	-4,20543	-69,93281	CAH-074		x
Euglossa	bursigera	Panama	9,15210	-79,84648	268	x	x
Euglossa	bursigera	Panama	9,15210	-79,84648	274	x	x

Euglossa	bursigera	Panama	9,15210	-79,84648	303		x
Euglossa	bursigera	Costa Rica	9,62626	-82,84993	1679	x	x
Euglossa	bursigera	Costa Rica	8,70342	-83,18755	1658		x
Euglossa	bursigera	Panama	9,55390	-79,65580	1181	x	
Euglossa	bursigera	Panama	9,55390	-79,65580	1184	x	
Euglossa	bursigera	Panama	9,12851	-79,71529	511	x	
Euglossa	bursigera	Colombia	5,58895	-75,86719	CAH-008	x	
Euglossa	bursigera	Colombia	5,58895	-75,86719	CAH-014		x
Euglossa	bursigera	Colombia	5,58895	-75,86719	CAH-027		x
Euglossa	bursigera	Panama	8,85991	-79,87868	345	x	x
Euglossa	bursigera	Panama	8,85991	-79,87868	1187	x	
Euglossa	bursigera	Panama	8,85991	-79,87868	1188	x	
Euglossa	chalybeata	Ecuador	-0,79913	-75,51029	CAH-272		x
Euglossa	chalybeata	Colombia	-4,20543	-69,93281	CAH-126		x
Euglossa	chalybeata	Colombia	-4,20543	-69,93281	CAH-127		x
Euglossa	chalybeata	Guyane	4,54893	-52,49186	1317	x	x
Euglossa	chalybeata	Guyane	4,78401	-52,43075	1247	x	
Euglossa	chalybeata	Guyane	4,78401	-52,43075	1251	x	
Euglossa	chalybeata	Guyane	4,78401	-52,43075	1260	x	
Euglossa	chalybeata	Guyane	4,78401	-52,43075	1367	x	x
Euglossa	chalybeata	Guyane	4,78401	-52,43075	1370	x	x
Euglossa	chalybeata	Guyane	4,78401	-52,43075	1384	x	x
Euglossa	chalybeata	Guyane	4,78401	-52,43075	1385	x	x
Euglossa	chalybeata	Guyane	4,78401	-52,43075	1389		x
Euglossa	chalybeata	Guyane	4,78401	-52,43075	1562	x	
Euglossa	chalybeata	Guyane	4,78401	-52,43075	1565	x	
Euglossa	chalybeata	Guyane	4,53861	-52,13694	1271		x
Euglossa	chalybeata	Guyane	4,53861	-52,13694	1274		x
Euglossa	chalybeata	Guyane	4,53861	-52,13694	1275	x	x
Euglossa	chalybeata	Guyane	4,53861	-52,13694	1278		x
Euglossa	chalybeata	Guyane	5,09747	-52,69425	1451		x
Euglossa	chalybeata	Guyane	5,09747	-52,69425	1462		x
Euglossa	chalybeata	Guyane	5,09747	-52,69425	1463		x
Euglossa	chalybeata	Guyane	5,09747	-52,69425	1471	x	x
Euglossa	chalybeata	Guyane	4,83111	-52,44389	1349		x
Euglossa	chalybeata	Guyane	4,83111	-52,44389	1355	x	x
Euglossa	chalybeata	Colombia	5,58895	-75,86719	CAH-031		x
Euglossa	chalybeata	Colombia	5,58895	-75,86719	CAH-040		x
Euglossa	chalybeata	Guyane	5,15626	-52,88575	1391		x
Euglossa	chalybeata	Guyane	5,15626	-52,88575	1398	x	x
Euglossa	chalybeata	Guyane	5,15626	-52,88575	1399		x
Euglossa	chalybeata	Guyane	5,15626	-52,88575	1407	x	x
Euglossa	chalybeata	Guyane	5,15626	-52,88575	1417	x	x
Euglossa	chalybeata	Guyane	5,15626	-52,88575	1419	x	x
Euglossa	chalybeata	Colombia	-4,20543	-69,93281	CAH-079	x	x
Euglossa	chalybeata	Colombia	-4,20543	-69,93281	CAH-121	x	x
Euglossa	chalybeata	Colombia	-4,20543	-69,93281	CAH-082	x	x

Euglossa	championi	Panama	9,15210	-79,84648	270		x
Euglossa	championi	Colombia	6,08334	-77,41670	CAH-143		x
Euglossa	championi	Colombia	6,08334	-77,41670	CAH-154	x	x
Euglossa	championi	Colombia	6,08334	-77,41670	CAH-182		x
Euglossa	championi	Colombia	6,08334	-77,41670	CAH-213		x
Euglossa	championi	Colombia	6,08334	-77,41670	CAH-214	x	x
Euglossa	championi	Colombia	6,08334	-77,41670	CAH-219	x	x
Euglossa	championi	Costa Rica	8,70342	-83,18755	465		x
Euglossa	championi	Costa Rica	8,70342	-83,18755	466	x	x
Euglossa	championi	Costa Rica	8,70342	-83,18755	1616	x	x
Euglossa	championi	Costa Rica	8,70342	-83,18755	1626	x	x
Euglossa	championi	Costa Rica	8,70342	-83,18755	1635	x	x
Euglossa	championi	Costa Rica	8,70342	-83,18755	1639		x
Euglossa	championi	Costa Rica	8,70342	-83,18755	1642		x
Euglossa	championi	Costa Rica	8,70342	-83,18755	1643		x
Euglossa	championi	Costa Rica	8,70342	-83,18755	1664	x	x
Euglossa	championi	Panama	8,85991	-79,87868	355	x	x
Euglossa	championi	Panama	8,85991	-79,87868	366	x	
Euglossa	championi	Panama	8,85991	-79,87868	1189	x	
Euglossa	chlorina	Ecuador	-0,79913	-75,51029	CAH-257		x
Euglossa	chlorina	Colombia	-4,20543	-69,93281	CAH-085		x
Euglossa	chlorina	Colombia	-4,20543	-69,93281	CAH-097		x
Euglossa	chlorina	Guyane	4,78401	-52,43075	1363	x	x
Euglossa	chlorina	Guyane	4,78401	-52,43075	1364	x	
Euglossa	chlorina	Guyane	4,30968	-52,13418	1490		x
Euglossa	chlorina	Guyane	4,53861	-52,13694	1273	x	x
Euglossa	chlorina	Guyane	5,09747	-52,69425	1441		x
Euglossa	chlorina	Guyane	5,09747	-52,69425	1442		x
Euglossa	chlorina	Guyane	5,09747	-52,69425	1458	x	x
Euglossa	chlorina	Guyane	5,09747	-52,69425	1477	x	x
Euglossa	chlorina	Guyane	5,15626	-52,88575	1401	x	
Euglossa	chlorina	Guyane	5,15626	-52,88575	1410	x	
Euglossa	chlorina	Guyane	5,15626	-52,88575	1411	x	x
Euglossa	chlorina	Guyane	5,15626	-52,88575	1418	x	x
Euglossa	chlorina	Guyane	5,15626	-52,88575	1421	x	x
Euglossa	cognata	Ecuador	-0,79913	-75,51029	CAH-305	x	x
Euglossa	cognata	Ecuador	-0,79913	-75,51029	CAH-319	x	x
Euglossa	cognata	Colombia	-4,20543	-69,93281	CAH-065		x
Euglossa	cognata	Colombia	-4,20543	-69,93281	CAH-072		x
Euglossa	cognata	Colombia	-4,20543	-69,93281	CAH-083		x
Euglossa	cognata	Colombia	-4,20543	-69,93281	CAH-086		x
Euglossa	cognata	Colombia	6,08334	-77,41670	CAH-205		x
Euglossa	cognata	Colombia	6,08334	-77,41670	CAH-208		x
Euglossa	cognata	Guyane	4,78401	-52,43075	1387		x
Euglossa	cognata	Costa Rica	8,70342	-83,18755	464	x	
Euglossa	cognata	Costa Rica	8,70342	-83,18755	1663	x	
Euglossa	cognata	Guyane	4,53861	-52,13694	1287	x	x

Euglossa	cognata	Guyane	5,09747	-52,69425	1454		x
Euglossa	cognata	Guyane	5,09747	-52,69425	1456	x	x
Euglossa	cognata	Guyane	5,09747	-52,69425	1473		x
Euglossa	cognata	Guyane	5,09747	-52,69425	1474		x
Euglossa	cognata	Panama	9,12851	-79,71529	1217	x	
Euglossa	cognata	Colombia	5,58895	-75,86719	CAH-033	x	x
Euglossa	cognata	Guyane	5,15626	-52,88575	1396	x	x
Euglossa	cordata	Guyane	4,78401	-52,43075	1231	x	
Euglossa	cordata	Guyane	4,78401	-52,43075	1368	x	x
Euglossa	cordata	Guyane	4,78401	-52,43075	1432	x	x
Euglossa	cordata	Guyane	4,78401	-52,43075	1530	x	
Euglossa	cordata	Guyane	4,78401	-52,43075	1554	x	
Euglossa	cordata	Guyane	4,78401	-52,43075	1566	x	
Euglossa	cordata	Guyane	4,53861	-52,13694	1279	x	
Euglossa	cordata	Guyane	5,09747	-52,69425	1455		x
Euglossa	cordata	Guyane	5,09747	-52,69425	1457	x	x
Euglossa	cordata	Guyane	5,09747	-52,69425	1497	x	x
Euglossa	cordata	Guyane	5,09747	-52,69425	1499	x	x
Euglossa	cordata	Guyane	5,09747	-52,69425	1502	x	x
Euglossa	cordata	Guyane	5,09747	-52,69425	1517	x	x
Euglossa	cordata	Colombia	5,58895	-75,86719	CAH-006		x
Euglossa	crassipunctata	Panama	9,15210	-79,84648	267		x
Euglossa	crassipunctata	Panama	9,15210	-79,84648	292		x
Euglossa	crassipunctata	Colombia	6,08334	-77,41670	CAH-179	x	x
Euglossa	crassipunctata	Colombia	6,08334	-77,41670	CAH-195	x	x
Euglossa	crassipunctata	Colombia	6,08334	-77,41670	CAH-210		x
Euglossa	crassipunctata	Colombia	6,08334	-77,41670	CAH-211	x	x
Euglossa	crassipunctata	Colombia	6,08334	-77,41670	CAH-212	x	x
Euglossa	crassipunctata	Colombia	6,08334	-77,41670	CAH-222	x	x
Euglossa	crassipunctata	Colombia	6,08334	-77,41670	CAH-223	x	x
Euglossa	crassipunctata	Colombia	6,08334	-77,41670	CAH-226	x	x
Euglossa	crassipunctata	Colombia	6,08334	-77,41670	CAH-234	x	x
Euglossa	crassipunctata	Guyane	4,78401	-52,43075	1371		x
Euglossa	crassipunctata	Costa Rica	8,70342	-83,18755	1599		x
Euglossa	crassipunctata	Colombia	5,58895	-75,86719	CAH-045		x
Euglossa	cyanaspis	Panama	9,12851	-79,71529	513	x	
Euglossa	cyanaspis	Panama	8,85991	-79,87868	1191	x	
Euglossa	cyanaspis	Panama	8,85991	-79,87868	1195	x	
Euglossa	cyanaspis	Panama	8,85991	-79,87868	1193	x	
Euglossa	cyanura	Mexico	17,07920	-97,86740	709	x	x
Euglossa	cyanura	Mexico	17,07920	-97,86740	988	x	
Euglossa	cyanura	Mexico	18,99228	-96,13856	63	x	
Euglossa	cyanura	Mexico	18,99228	-96,13856	64	x	
Euglossa	cyanura	Mexico	18,99228	-96,13856	65		x
Euglossa	cyanura	Mexico	18,99228	-96,13856	66		x
Euglossa	cyanura	Mexico	18,99228	-96,13856	67	x	x
Euglossa	cyanura	Mexico	18,99228	-96,13856	68	x	

Euglossa	cyanura	Mexico	18,99228	-96,13856	70	x	
Euglossa	cyanura	Mexico	18,99228	-96,13856	549	x	
Euglossa	cyanura	Mexico	20,62009	-97,68576	588	x	x
Euglossa	cyanura	Mexico	20,62009	-97,68576	589	x	
Euglossa	cyanura	Mexico	20,62009	-97,68576	590	x	
Euglossa	cyanura	Mexico	20,62009	-97,68576	591	x	
Euglossa	cyanura	Mexico	20,62009	-97,68576	592	x	
Euglossa	cyanura	Mexico	20,62009	-97,68576	599	x	
Euglossa	cyanura	Mexico	20,62009	-97,68576	758	x	
Euglossa	cyanura	Mexico	20,62009	-97,68576	762	x	
Euglossa	cyanura	Mexico	17,04520	-96,75491	225	x	
Euglossa	cyanura	Panama	8,85991	-79,87868	348	x	x
Euglossa	cybelia	Ecuador	-0,79913	-75,51029	CAH-295	x	x
Euglossa	cybelia	Ecuador	-0,79913	-75,51029	CAH-306	x	x
Euglossa	cybelia	Ecuador	-0,79913	-75,51029	CAH-316		x
Euglossa	cybelia	Colombia	-4,20543	-69,93281	CAH-088		x
Euglossa	cybelia	Panama	9,15210	-79,84648	310		x
Euglossa	cybelia	Panama	9,15210	-79,84648	312	x	x
Euglossa	cybelia	Costa Rica	8,70342	-83,18755	1600		x
Euglossa	cybelia	Costa Rica	8,70342	-83,18755	1603	x	
Euglossa	cybelia	Costa Rica	8,70342	-83,18755	1620	x	
Euglossa	cybelia	Costa Rica	8,70342	-83,18755	1631	x	
Euglossa	cybelia	Costa Rica	8,70342	-83,18755	1637	x	x
Euglossa	cybelia	Costa Rica	8,70342	-83,18755	1640	x	x
Euglossa	cybelia	Costa Rica	8,70342	-83,18755	1661	x	
Euglossa	cybelia	Costa Rica	8,70342	-83,18755	1667	x	x
Euglossa	cybelia	Colombia	5,58895	-75,86719	CAH-010		x
Euglossa	cybelia	Colombia	5,58895	-75,86719	CAH-023		x
Euglossa	cybelia	Panama	8,85991	-79,87868	1190	x	
Euglossa	cybelia	Costa Rica	8,70342	-83,18755	474	x	
Euglossa	cybelia	Panama	9,12851	-79,71529	512	x	
Euglossa	cybelia	Costa Rica	10,39896	-84,13506	428		x
Euglossa	cybelia	Costa Rica	10,39896	-84,13506	429	x	x
Euglossa	cybelia	Costa Rica	10,39896	-84,13506	430	x	x
Euglossa	cybelia	Costa Rica	10,39896	-84,13506	431	x	x
Euglossa	decorata	Ecuador	-0,79913	-75,51029	CAH-292	x	x
Euglossa	decorata	Ecuador	-0,79913	-75,51029	CAH-293	x	x
Euglossa	decorata	Ecuador	-0,79913	-75,51029	CAH-297	x	x
Euglossa	decorata	Ecuador	-0,79913	-75,51029	CAH-298	x	
Euglossa	decorata	Colombia	-4,20543	-69,93281	CAH-080	x	x
Euglossa	decorata	Colombia	-4,20543	-69,93281	CAH-122	x	x
Euglossa	decorata	Colombia	-4,20543	-69,93281	CAH-123	x	x
Euglossa	decorata	Guyane	5,09747	-52,69425	1466	x	x
Euglossa	decorata	Guyane	5,09747	-52,69425	1468	x	x
Euglossa	decorata	Guyane	5,09747	-52,69425	1524	x	x
Euglossa	despecta	Ecuador	-0,79913	-75,51029	CAH-324	x	x
Euglossa	despecta	Panama	9,15210	-79,84648	276	x	x

Euglossa	despecta	Panama	9,15210	-79,84648	322	x	x
Euglossa	despecta	Costa Rica	9,62626	-82,84993	1684	x	x
Euglossa	despecta	Colombia	6,08334	-77,41670	CAH-209		x
Euglossa	despecta	Colombia	6,08334	-77,41670	CAH-216		x
Euglossa	despecta	Colombia	6,08334	-77,41670	CAH-218	x	x
Euglossa	despecta	Colombia	6,08334	-77,41670	CAH-232		x
Euglossa	despecta	Costa Rica	8,70342	-83,18755	1617		x
Euglossa	despecta	Costa Rica	8,70342	-83,18755	1618		x
Euglossa	despecta	Costa Rica	8,70342	-83,18755	1622		x
Euglossa	despecta	Costa Rica	8,70342	-83,18755	1660		x
Euglossa	despecta	Panama	9,55390	-79,65580	1185	x	
Euglossa	despecta	Panama	9,12851	-79,71529	515	x	
Euglossa	despecta	Panama	9,12851	-79,71529	1219	x	
Euglossa	despecta	Colombia	5,58895	-75,86719	CAH-017		x
Euglossa	despecta	Colombia	5,58895	-75,86719	CAH-038		x
Euglossa	despecta	Colombia	5,58895	-75,86719	CAH-042		x
Euglossa	despecta	Colombia	5,58895	-75,86719	CAH-044		x
Euglossa	dissimula	Panama	9,15210	-79,84648	271		x
Euglossa	dissimula	Panama	9,15210	-79,84648	325	x	x
Euglossa	dissimula	Panama	9,12851	-79,71529	509	x	x
Euglossa	dissimula	Panama	9,12851	-79,71529	514	x	x
Euglossa	dissimula	Panama	8,85991	-79,87868	346	x	x
Euglossa	dodsoni	Panama	9,15210	-79,84648	295		x
Euglossa	dodsoni	Costa Rica	9,62626	-82,84993	1672		x
Euglossa	dodsoni	Colombia	6,08334	-77,41670	CAH-181		x
Euglossa	dodsoni	Costa Rica	8,70342	-83,18755	489	x	x
Euglossa	dodsoni	Costa Rica	8,70342	-83,18755	497	x	x
Euglossa	dodsoni	Costa Rica	8,70342	-83,18755	498	x	
Euglossa	dodsoni	Costa Rica	8,70342	-83,18755	500	x	
Euglossa	dodsoni	Costa Rica	8,70342	-83,18755	505	x	
Euglossa	dodsoni	Costa Rica	8,70342	-83,18755	1621	x	x
Euglossa	dodsoni	Costa Rica	8,70342	-83,18755	1629	x	x
Euglossa	dodsoni	Costa Rica	8,70342	-83,18755	1638	x	x
Euglossa	dodsoni	Costa Rica	8,70342	-83,18755	1641	x	x
Euglossa	dodsoni	Costa Rica	10,39896	-84,13506	424	x	x
Euglossa	dodsoni	Costa Rica	10,39896	-84,13506	425	x	x
Euglossa	dodsoni	Costa Rica	10,39896	-84,13506	448	x	
Euglossa	erythrochlora	Costa Rica	8,70342	-83,18755	475	x	x
Euglossa	erythrochlora	Costa Rica	8,70342	-83,18755	476		x
Euglossa	erythrochlora	Costa Rica	8,70342	-83,18755	477	x	x
Euglossa	erythrochlora	Costa Rica	8,70342	-83,18755	479	x	
Euglossa	erythrochlora	Costa Rica	8,70342	-83,18755	481	x	
Euglossa	erythrochlora	Costa Rica	8,70342	-83,18755	484	x	
Euglossa	flammea	Costa Rica	8,70342	-83,18755	1628		x
Euglossa	flammea	Costa Rica	8,70342	-83,18755	1632		x
Euglossa	flammea	Costa Rica	8,70342	-83,18755	1646		x
Euglossa	flammea	Costa Rica	8,70342	-83,18755	1662	x	x

Euglossa	flammea	Costa Rica	8,70342	-83,18755	1665		x
Euglossa	flammea	Costa Rica	8,70342	-83,18755	482	x	x
Euglossa	flammea	Costa Rica	8,70342	-83,18755	483	x	
Euglossa	flammea	Costa Rica	8,70342	-83,18755	492	x	x
Euglossa	flammea	Costa Rica	8,70342	-83,18755	493	x	x
Euglossa	flammea	Costa Rica	8,70342	-83,18755	494	x	
Euglossa	fuscifrons	Ecuador	-0,79913	-75,51029	CAH-242	x	x
Euglossa	fuscifrons	Ecuador	-0,79913	-75,51029	CAH-255	x	x
Euglossa	fuscifrons	Ecuador	-0,79913	-75,51029	CAH-280	x	x
Euglossa	fuscifrons	Ecuador	-0,79913	-75,51029	CAH-282	x	x
Euglossa	fuscifrons	Ecuador	-0,79913	-75,51029	CAH-284	x	x
Euglossa	fuscifrons	Ecuador	-0,79913	-75,51029	CAH-287		x
Euglossa	fuscifrons	Ecuador	-0,79913	-75,51029	CAH-294		x
Euglossa	fuscifrons	Ecuador	-0,79913	-75,51029	CAH-301	x	x
Euglossa	fuscifrons	Ecuador	-0,79913	-75,51029	CAH-312	x	x
Euglossa	fuscifrons	Ecuador	-0,79913	-75,51029	CAH-317		x
Euglossa	fuscifrons	Ecuador	-0,79913	-75,51029	CAH-318	x	x
Euglossa	fuscifrons	Ecuador	-0,79913	-75,51029	CAH-323	x	x
Euglossa	fuscifrons	Ecuador	-0,79913	-75,51029	CAH-326	x	x
Euglossa	fuscifrons	Ecuador	-0,79913	-75,51029	CAH-328	x	x
Euglossa	gorgonensis	Panama	9,15210	-79,84648	273	x	x
Euglossa	gorgonensis	Colombia	6,08334	-77,41670	CAH-159		x
Euglossa	gorgonensis	Colombia	6,08334	-77,41670	CAH-163		x
Euglossa	gorgonensis	Colombia	6,08334	-77,41670	CAH-165		x
Euglossa	gorgonensis	Colombia	6,08334	-77,41670	CAH-166	x	x
Euglossa	gorgonensis	Colombia	6,08334	-77,41670	CAH-167	x	x
Euglossa	gorgonensis	Colombia	6,08334	-77,41670	CAH-168	x	x
Euglossa	gorgonensis	Colombia	6,08334	-77,41670	CAH-225	x	x
Euglossa	gorgonensis	Costa Rica	8,70342	-83,18755	1614		x
Euglossa	gorgonensis	Costa Rica	8,70342	-83,18755	1644		x
Euglossa	gorgonensis	Colombia	5,58895	-75,86719	CAH-009	x	
Euglossa	gorgonensis	Colombia	5,58895	-75,86719	CAH-035		x
Euglossa	gorgonensis	Ecuador	-0,79913	-75,51029	CAH-245		x
Euglossa	hansoni	Costa Rica	9,62626	-82,84993	1674		x
Euglossa	hansoni	Costa Rica	9,62626	-82,84993	1676	x	
Euglossa	hansoni	Costa Rica	9,62626	-82,84993	1681		x
Euglossa	hansoni	Costa Rica	9,62626	-82,84993	1685		x
Euglossa	hansoni	Colombia	6,08334	-77,41670	CAH-161	x	x
Euglossa	hansoni	Colombia	6,08334	-77,41670	CAH-171	x	x
Euglossa	hansoni	Colombia	6,08334	-77,41670	CAH-172	x	x
Euglossa	hansoni	Colombia	6,08334	-77,41670	CAH-173		x
Euglossa	hansoni	Colombia	6,08334	-77,41670	CAH-175		x
Euglossa	hansoni	Colombia	6,08334	-77,41670	CAH-176		x
Euglossa	hansoni	Colombia	6,08334	-77,41670	CAH-237	x	x
Euglossa	hansoni	Colombia	6,08334	-77,41670	CAH-239	x	x
Euglossa	hansoni	Costa Rica	8,70342	-83,18755	507	x	
Euglossa	hansoni	Costa Rica	8,70342	-83,18755	1615		x

Euglossa	hansoni	Panama	9,55390	-79,65580	517	x	
Euglossa	hansoni	Panama	9,55390	-79,65580	519	x	
Euglossa	hansoni	Panama	8,85991	-79,87868	335		x
Euglossa	hansoni	Panama	8,85991	-79,87868	336		x
Euglossa	hansoni	Panama	8,85991	-79,87868	337	x	x
Euglossa	hansoni	Panama	8,85991	-79,87868	338	x	x
Euglossa	hansoni	Panama	8,85991	-79,87868	339	x	x
Euglossa	hansoni	Panama	8,85991	-79,87868	340	x	
Euglossa	hansoni	Panama	8,85991	-79,87868	341	x	
Euglossa	hansoni	Panama	8,85991	-79,87868	342	x	
Euglossa	hansoni	Panama	8,85991	-79,87868	1206	x	
Euglossa	hansoni	Costa Rica	10,39896	-84,13506	446	x	x
Euglossa	hansoni	Costa Rica	10,39896	-84,13506	447	x	
Euglossa	hemichlora	Ecuador	-0,79913	-75,51029	CAH-261		x
Euglossa	hemichlora	Ecuador	-0,79913	-75,51029	CAH-283	x	x
Euglossa	hemichlora	Panama	9,15210	-79,84648	275	x	x
Euglossa	hemichlora	Panama	9,15210	-79,84648	305	x	x
Euglossa	hemichlora	Panama	9,15210	-79,84648	306	x	x
Euglossa	hemichlora	Colombia	6,08334	-77,41670	CAH-169		x
Euglossa	hemichlora	Costa Rica	8,70342	-83,18755	1627	x	x
Euglossa	hemichlora	Costa Rica	8,70342	-83,18755	1630	x	x
Euglossa	hemichlora	Costa Rica	8,70342	-83,18755	1655	x	x
Euglossa	hemichlora	Costa Rica	8,70342	-83,18755	1659		x
Euglossa	hemichlora	Mexico	16,60344	-90,91443	23	x	
Euglossa	hemichlora	Mexico	17,51020	-91,98104	8	x	
Euglossa	hemichlora	Panama	8,85991	-79,87868	353	x	
Euglossa	hemichlora	Panama	9,15210	-79,84648	272		x
Euglossa	heterostica	Panama	8,85991	-79,87868	1194	x	
Euglossa	heterosticta	Panama	9,15210	-79,84648	324		x
Euglossa	heterosticta	Colombia	6,08334	-77,41670	CAH-158		x
Euglossa	heterosticta	Colombia	6,08334	-77,41670	CAH-236	x	
Euglossa	heterosticta	Costa Rica	8,70342	-83,18755	486	x	
Euglossa	heterosticta	Costa Rica	8,70342	-83,18755	501	x	
Euglossa	heterosticta	Costa Rica	8,70342	-83,18755	502	x	x
Euglossa	heterosticta	Costa Rica	8,70342	-83,18755	503	x	
Euglossa	heterosticta	Costa Rica	8,70342	-83,18755	1601		x
Euglossa	heterosticta	Costa Rica	8,70342	-83,18755	1647		x
Euglossa	heterosticta	Costa Rica	8,70342	-83,18755	1656		x
Euglossa	heterosticta	Costa Rica	8,70342	-83,18755	1669		x
Euglossa	heterosticta	Mexico	16,60344	-90,91443	20	x	
Euglossa	heterosticta	Mexico	18,99228	-96,13856	1025	x	x
Euglossa	heterosticta	Panama	9,55390	-79,65580	1180	x	
Euglossa	heterosticta	Mexico	17,51020	-91,98104	51	x	x
Euglossa	heterosticta	Mexico	17,51020	-91,98104	52	x	x
Euglossa	heterosticta	Panama	8,85991	-79,87868	347	x	x
Euglossa	ignita	Ecuador	-0,79913	-75,51029	CAH-271	x	x
Euglossa	ignita	Colombia	-4,20543	-69,93281	CAH-062		x

Euglossa	ignita	Colombia	-4,20543	-69,93281	CAH-068	x	x
Euglossa	ignita	Colombia	-4,20543	-69,93281	CAH-069		x
Euglossa	ignita	Colombia	-4,20543	-69,93281	CAH-073	x	x
Euglossa	ignita	Colombia	-4,20543	-69,93281	CAH-075	x	x
Euglossa	ignita	Colombia	-4,20543	-69,93281	CAH-076	x	x
Euglossa	ignita	Colombia	-4,20543	-69,93281	CAH-078	x	x
Euglossa	ignita	Colombia	-4,20543	-69,93281	CAH-092	x	x
Euglossa	ignita	Colombia	-4,20543	-69,93281	CAH-095	x	x
Euglossa	ignita	Colombia	-4,20543	-69,93281	CAH-098		x
Euglossa	ignita	Colombia	-4,20543	-69,93281	CAH-107		x
Euglossa	ignita	Colombia	-4,20543	-69,93281	CAH-117	x	x
Euglossa	ignita	Colombia	-4,20543	-69,93281	CAH-119		x
Euglossa	ignita	Colombia	-4,20543	-69,93281	CAH-120		x
Euglossa	ignita	Colombia	-4,20543	-69,93281	CAH-124	x	x
Euglossa	ignita	Colombia	-4,20543	-69,93281	CAH-125		x
Euglossa	ignita	Costa Rica	9,62626	-82,84993	1683		x
Euglossa	ignita	Guyane	4,54893	-52,49186	1296		x
Euglossa	ignita	Guyane	4,54893	-52,49186	1309		x
Euglossa	ignita	Guyane	4,54893	-52,49186	1318		x
Euglossa	ignita	Guyane	4,54893	-52,49186	1332		x
Euglossa	ignita	Guyane	4,54893	-52,49186	1333	x	x
Euglossa	ignita	Colombia	6,08334	-77,41670	CAH-140C	x	x
Euglossa	ignita	Colombia	6,08334	-77,41670	CAH-141		x
Euglossa	ignita	Colombia	6,08334	-77,41670	CAH-142	x	x
Euglossa	ignita	Colombia	6,08334	-77,41670	CAH-145	x	x
Euglossa	ignita	Colombia	6,08334	-77,41670	CAH-149		x
Euglossa	ignita	Colombia	6,08334	-77,41670	CAH-150		x
Euglossa	ignita	Colombia	6,08334	-77,41670	CAH-164	x	x
Euglossa	ignita	Guyane	4,78401	-52,43075	1223	x	
Euglossa	ignita	Guyane	4,78401	-52,43075	1224	x	
Euglossa	ignita	Guyane	4,78401	-52,43075	1225	x	
Euglossa	ignita	Guyane	4,78401	-52,43075	1232	x	
Euglossa	ignita	Guyane	4,78401	-52,43075	1248	x	
Euglossa	ignita	Guyane	4,78401	-52,43075	1258	x	
Euglossa	ignita	Guyane	4,78401	-52,43075	1561	x	
Euglossa	ignita	Guyane	4,53861	-52,13694	1280	x	x
Euglossa	ignita	Guyane	5,09747	-52,69425	1443		x
Euglossa	ignita	Guyane	5,09747	-52,69425	1453		x
Euglossa	ignita	Guyane	4,83111	-52,44389	1344		x
Euglossa	ignita	Colombia	5,58895	-75,86719	CAH-037		x
Euglossa	ignita	Colombia	5,58895	-75,86719	CAH-039	x	x
Euglossa	ignita	Costa Rica	10,39896	-84,13506	450	x	x
Euglossa	ignita	Colombia	-4,20543	-69,93281	CAH-057	x	x
Euglossa	ignita	Colombia	-4,20543	-69,93281	CAH-081	x	x
Euglossa	ignita	Colombia	-4,20543	-69,93281	CAH-084	x	x
Euglossa	igniventris	Panama	9,15210	-79,84648	321	x	x
Euglossa	igniventris	Panama	9,15210	-79,84648	323	x	x

Euglossa	igniventris	Colombia	6,08334	-77,41670	CAH-160		x
Euglossa	igniventris	Panama	9,55390	-79,65580	1183	x	
Euglossa	imperialis	Ecuador	-0,79913	-75,51029	CAH-248	x	x
Euglossa	imperialis	Ecuador	-0,79913	-75,51029	CAH-268	x	x
Euglossa	imperialis	Ecuador	-0,79913	-75,51029	CAH-273	x	x
Euglossa	imperialis	Ecuador	-0,79913	-75,51029	CAH-274	x	x
Euglossa	imperialis	Ecuador	-0,79913	-75,51029	CAH-276	x	x
Euglossa	imperialis	Ecuador	-0,79913	-75,51029	CAH-285	x	x
Euglossa	imperialis	Ecuador	-0,79913	-75,51029	CAH-322	x	x
Euglossa	imperialis	Ecuador	-0,79913	-75,51029	CAH-327	x	x
Euglossa	imperialis	Ecuador	-0,79913	-75,51029	CAH-329	x	x
Euglossa	imperialis	Panama	9,15210	-79,84648	269		x
Euglossa	imperialis	Costa Rica	9,62626	-82,84993	1678		x
Euglossa	imperialis	Costa Rica	9,62626	-82,84993	1680		x
Euglossa	imperialis	Colombia	6,08334	-77,41670	CAH-146		x
Euglossa	imperialis	Colombia	6,08334	-77,41670	CAH-147		x
Euglossa	imperialis	Colombia	6,08334	-77,41670	CAH-148		x
Euglossa	imperialis	Colombia	6,08334	-77,41670	CAH-151		x
Euglossa	imperialis	Colombia	6,08334	-77,41670	CAH-152		x
Euglossa	imperialis	Colombia	6,08334	-77,41670	CAH-153		x
Euglossa	imperialis	Guyane	4,78401	-52,43075	1378		x
Euglossa	imperialis	Guyane	4,78401	-52,43075	1590	x	
Euglossa	imperialis	Guyane	4,78401	-52,43075	1592	x	
Euglossa	imperialis	Mexico	16,60344	-90,91443	31	x	
Euglossa	imperialis	Mexico	16,60344	-90,91443	32	x	
Euglossa	imperialis	Mexico	16,60344	-90,91443	33	x	
Euglossa	imperialis	Mexico	16,60344	-90,91443	34	x	
Euglossa	imperialis	Mexico	16,60344	-90,91443	35	x	
Euglossa	imperialis	Mexico	16,60344	-90,91443	36	x	
Euglossa	imperialis	Mexico	17,51020	-91,98104	14	x	
Euglossa	imperialis	Mexico	17,51020	-91,98104	15	x	
Euglossa	imperialis	Mexico	17,51020	-91,98104	16	x	
Euglossa	imperialis	Mexico	17,51020	-91,98104	17	x	
Euglossa	imperialis	Mexico	17,51020	-91,98104	18	x	
Euglossa	imperialis	Mexico	20,62009	-97,68576	755		x
Euglossa	imperialis	Colombia	5,58895	-75,86719	CAH-011		x
Euglossa	imperialis	Colombia	5,58895	-75,86719	CAH-012		x
Euglossa	imperialis	Colombia	5,58895	-75,86719	CAH-016		x
Euglossa	imperialis	Colombia	5,58895	-75,86719	CAH-019		x
Euglossa	imperialis	Colombia	5,58895	-75,86719	CAH-020		x
Euglossa	intersecta	Ecuador	-0,79913	-75,51029	CAH-265	x	x
Euglossa	intersecta	Ecuador	-0,79913	-75,51029	CAH-266	x	x
Euglossa	intersecta	Ecuador	-0,79913	-75,51029	CAH-270	x	x
Euglossa	intersecta	Ecuador	-0,79913	-75,51029	CAH-278	x	x
Euglossa	intersecta	Ecuador	-0,79913	-75,51029	CAH-303		x
Euglossa	intersecta	Colombia	-4,20543	-69,93281	CAH-047		x
Euglossa	intersecta	Colombia	-4,20543	-69,93281	CAH-051		x

Euglossa	intersecta	Colombia	-4,20543	-69,93281	CAH-054		x
Euglossa	intersecta	Colombia	-4,20543	-69,93281	CAH-055		x
Euglossa	intersecta	Colombia	-4,20543	-69,93281	CAH-112	x	x
Euglossa	intersecta	Guyane	4,54893	-52,49186	1298	x	x
Euglossa	intersecta	Guyane	4,78401	-52,43075	1243	x	
Euglossa	intersecta	Guyane	4,78401	-52,43075	1255	x	
Euglossa	intersecta	Guyane	4,78401	-52,43075	1427	x	x
Euglossa	intersecta	Guyane	4,78401	-52,43075	1430	x	x
Euglossa	intersecta	Guyane	4,78401	-52,43075	1431	x	x
Euglossa	intersecta	Guyane	4,78401	-52,43075	1531	x	
Euglossa	intersecta	Guyane	4,78401	-52,43075	1555	x	
Euglossa	intersecta	Guyane	4,78401	-52,43075	1583	x	
Euglossa	intersecta	Guyane	4,78401	-52,43075	1594	x	
Euglossa	intersecta	Guyane	5,09747	-52,69425	1483	x	
Euglossa	intersecta	Guyane	5,09747	-52,69425	1484	x	
Euglossa	intersecta	Guyane	5,09747	-52,69425	1485	x	
Euglossa	intersecta	Guyane	5,09747	-52,69425	1486	x	
Euglossa	laevicincta	Guyane	4,54893	-52,49186	1294	x	x
Euglossa	laevicincta	Guyane	4,78401	-52,43075	1359		x
Euglossa	laevicincta	Guyane	4,78401	-52,43075	1426		x
Euglossa	laevicincta	Guyane	4,78401	-52,43075	1581	x	
Euglossa	laevicincta	Guyane	5,09747	-52,69425	1464		x
Euglossa	laevicincta	Guyane	5,09747	-52,69425	1470		x
Euglossa	laevicincta	Guyane	5,09747	-52,69425	1505	x	x
Euglossa	laevicincta	Guyane	4,83111	-52,44389	1352	x	x
Euglossa	liopoda	Ecuador	-0,79913	-75,51029	CAH-288	x	x
Euglossa	liopoda	Colombia	-4,20543	-69,93281	CAH-060	x	x
Euglossa	liopoda	Colombia	-4,20543	-69,93281	CAH-129	x	x
Euglossa	liopoda	Colombia	-4,20543	-69,93281	CAH-132		x
Euglossa	liopoda	Guyane	4,78401	-52,43075	1365	x	x
Euglossa	liopoda	Guyane	5,09747	-52,69425	1472	x	x
Euglossa	liopoda	Colombia	5,58895	-75,86719	CAH-015	x	
Euglossa	liopoda	Colombia	5,58895	-75,86719	CAH-022		x
Euglossa	lugubris	Colombia	-4,20543	-69,93281	CAH-067		x
Euglossa	mixta	Ecuador	-0,79913	-75,51029	CAH-252	x	x
Euglossa	mixta	Ecuador	-0,79913	-75,51029	CAH-253	x	x
Euglossa	mixta	Ecuador	-0,79913	-75,51029	CAH-259	x	x
Euglossa	mixta	Ecuador	-0,79913	-75,51029	CAH-269	x	x
Euglossa	mixta	Ecuador	-0,79913	-75,51029	CAH-289	x	x
Euglossa	mixta	Ecuador	-0,79913	-75,51029	CAH-311		x
Euglossa	mixta	Ecuador	-0,79913	-75,51029	CAH-315	x	x
Euglossa	mixta	Colombia	-4,20543	-69,93281	CAH-052		x
Euglossa	mixta	Colombia	-4,20543	-69,93281	CAH-053		x
Euglossa	mixta	Colombia	-4,20543	-69,93281	CAH-063		x
Euglossa	mixta	Colombia	-4,20543	-69,93281	CAH-064	x	x
Euglossa	mixta	Colombia	-4,20543	-69,93281	CAH-066		x
Euglossa	mixta	Colombia	-4,20543	-69,93281	CAH-077		x

Euglossa	mixta	Colombia	-4,20543	-69,93281	CAH-089	x	x
Euglossa	mixta	Colombia	6,08334	-77,41670	CAH-193	x	x
Euglossa	mixta	Colombia	6,08334	-77,41670	CAH-194	x	x
Euglossa	mixta	Colombia	6,08334	-77,41670	CAH-206	x	x
Euglossa	mixta	Colombia	6,08334	-77,41670	CAH-207	x	x
Euglossa	mixta	Guyane	4,78401	-52,43075	1372	x	
Euglossa	mixta	Costa Rica	8,70342	-83,18755	508	x	
Euglossa	mixta	Costa Rica	8,70342	-83,18755	1623		x
Euglossa	mixta	Costa Rica	8,70342	-83,18755	1668		x
Euglossa	mixta	Mexico	16,60344	-90,91443	29	x	
Euglossa	mixta	Mexico	16,60344	-90,91443	30	x	
Euglossa	mixta	Mexico	18,99228	-96,13856	408	x	
Euglossa	mixta	Guyane	5,09747	-52,69425	1437	x	x
Euglossa	mixta	Mexico	18,64347	-95,09647	2	x	
Euglossa	mixta	Mexico	17,51020	-91,98104	10	x	
Euglossa	mixta	Mexico	17,04520	-96,75491	220		x
Euglossa	mixta	Colombia	5,58895	-75,86719	CAH-002	x	x
Euglossa	mixta	Colombia	5,58895	-75,86719	CAH-007	x	x
Euglossa	mixta	Colombia	5,58895	-75,86719	CAH-028		x
Euglossa	mixta	Colombia	5,58895	-75,86719	CAH-030	x	x
Euglossa	mixta	Colombia	5,58895	-75,86719	CAH-034		x
Euglossa	mixta	Colombia	5,58895	-75,86719	CAH-036		x
Euglossa	mixta	Panama	8,85991	-79,87868	352	x	
Euglossa	modestior	Guyane	4,54893	-52,49186	1306	x	x
Euglossa	modestior	Guyane	4,54893	-52,49186	1308	x	x
Euglossa	modestior	Guyane	4,54893	-52,49186	1311	x	x
Euglossa	modestior	Guyane	4,54893	-52,49186	1313	x	x
Euglossa	modestior	Guyane	4,54893	-52,49186	1315	x	x
Euglossa	modestior	Guyane	4,54893	-52,49186	1321	x	x
Euglossa	modestior	Colombia	6,08334	-77,41670	CAH-229	x	x
Euglossa	modestior	Guyane	4,78401	-52,43075	1240	x	
Euglossa	modestior	Guyane	4,78401	-52,43075	1382	x	x
Euglossa	modestior	Guyane	5,09747	-52,69425	1448	x	x
Euglossa	modestior	Colombia	5,58895	-75,86719	CAH-018		x
Euglossa	obtusa	Mexico	17,07920	-97,86740	651	x	
Euglossa	obtusa	Mexico	16,60344	-90,91443	21	x	
Euglossa	obtusa	Mexico	16,60344	-90,91443	22	x	
Euglossa	obtusa	Mexico	17,51020	-91,98104	7	x	
Euglossa	obtusa	Mexico	17,51020	-91,98104	9	x	
Euglossa	obtusa	Mexico	17,51020	-91,98104	53	x	x
Euglossa	obtusa	Mexico	17,51020	-91,98104	54	x	
Euglossa	obtusa	Mexico	17,51020	-91,98104	55	x	x
Euglossa	obtusa	Mexico	20,62009	-97,68576	605	x	
Euglossa	obtusa	Mexico	20,62009	-97,68576	643	x	x
Euglossa	obtusa	Mexico	17,04520	-96,75491	170	x	
Euglossa	obtusa	Mexico	17,04520	-96,75491	171	x	
Euglossa	obtusa	Mexico	17,04520	-96,75491	172	x	x

Euglossa	obtusa	Mexico	17,04520	-96,75491	173	x	
Euglossa	obtusa	Mexico	17,04520	-96,75491	174	x	
Euglossa	obtusa	Mexico	17,04520	-96,75491	175	x	
Euglossa	occidentalis	Ecuador	-0,79913	-75,51029	CAH-246	x	x
Euglossa	occidentalis	Ecuador	-0,79913	-75,51029	CAH-247	x	x
Euglossa	occidentalis	Ecuador	-0,79913	-75,51029	CAH-249	x	x
Euglossa	occidentalis	Ecuador	-0,79913	-75,51029	CAH-250		x
Euglossa	occidentalis	Ecuador	-0,79913	-75,51029	CAH-251	x	x
Euglossa	occidentalis	Ecuador	-0,79913	-75,51029	CAH-279	x	x
Euglossa	occidentalis	Ecuador	-0,79913	-75,51029	CAH-296	x	x
Euglossa	occidentalis	Ecuador	-0,79913	-75,51029	CAH-299	x	x
Euglossa	occidentalis	Ecuador	-0,79913	-75,51029	CAH-302	x	x
Euglossa	occidentalis	Ecuador	-0,79913	-75,51029	CAH-304	x	
Euglossa	occidentalis	Ecuador	-0,79913	-75,51029	CAH-325	x	x
Euglossa	occidentalis	Colombia	-4,20543	-69,93281	CAH-056		x
Euglossa	occidentalis	Colombia	-4,20543	-69,93281	CAH-070		x
Euglossa	occidentalis	Colombia	-4,20543	-69,93281	CAH-118		x
Euglossa	occidentalis	Colombia	-4,20543	-69,93281	CAH-087		x
Euglossa	orellana	Guyane	4,78401	-52,43075	1229	x	
Euglossa	orellana	Guyane	4,78401	-52,43075	1256	x	
Euglossa	orellana	Guyane	4,78401	-52,43075	1425	x	x
Euglossa	orellana	Guyane	4,53861	-52,13694	1286	x	
Euglossa	orellana	Guyane	5,09747	-52,69425	1506	x	x
Euglossa	orellana	Guyane	5,09747	-52,69425	1511	x	x
Euglossa	piliventris	Guyane	4,53861	-52,13694	2056		x
Euglossa	piliventris	Guyane	4,53861	-52,13694	2057		x
Euglossa	piliventris	Guyane	4,53861	-52,13694	2058		x
Euglossa	piliventris	Guyane	4,53861	-52,13694	2060		x
Euglossa	piliventris	Guyane	4,53861	-52,13694	2061		x
Euglossa	piliventris	Guyane	4,53861	-52,13694	2062		x
Euglossa	piliventris	Guyane	4,53861	-52,13694	2063		x
Euglossa	prasina	Colombia	-4,20543	-69,93281	CAH-090		x
Euglossa	prasina	Colombia	-4,20543	-69,93281	CAH-091	x	x
Euglossa	prasina	Guyane	4,54893	-52,49186	1297	x	x
Euglossa	prasina	Guyane	4,54893	-52,49186	1320		x
Euglossa	prasina	Guyane	4,78401	-52,43075	1249	x	
Euglossa	prasina	Guyane	4,78401	-52,43075	1252	x	
Euglossa	prasina	Guyane	4,78401	-52,43075	1559	x	
Euglossa	prasina	Guyane	4,78401	-52,43075	1573	x	
Euglossa	prasina	Guyane	4,53861	-52,13694	1285	x	
Euglossa	prasina	Guyane	4,83111	-52,44389	1341		x
Euglossa	prasina	Guyane	4,83111	-52,44389	1342		x
Euglossa	purpurea	Costa Rica	8,70342	-83,18755	457	x	
Euglossa	purpurea	Costa Rica	8,70342	-83,18755	458	x	
Euglossa	purpurea	Costa Rica	8,70342	-83,18755	459	x	
Euglossa	purpurea	Costa Rica	8,70342	-83,18755	460	x	
Euglossa	purpurea	Costa Rica	8,70342	-83,18755	461	x	

Euglossa	purpurea	Costa Rica	10,39896	-84,13506	420	x	x
Euglossa	purpurea	Costa Rica	10,39896	-84,13506	421	x	x
Euglossa	purpurea	Costa Rica	10,39896	-84,13506	422	x	x
Euglossa	purpurea	Costa Rica	10,39896	-84,13506	439	x	
Euglossa	purpurea	Costa Rica	10,39896	-84,13506	441	x	
Euglossa	purpurea	Costa Rica	10,39896	-84,13506	442	x	
Euglossa	purpurea	Costa Rica	10,39896	-84,13506	443	x	
Euglossa	sapphirina	Panama	9,15210	-79,84648	262	x	x
Euglossa	sapphirina	Panama	9,15210	-79,84648	263	x	x
Euglossa	sapphirina	Panama	9,15210	-79,84648	264	x	x
Euglossa	sapphirina	Panama	9,15210	-79,84648	265	x	x
Euglossa	sapphirina	Panama	9,15210	-79,84648	266	x	x
Euglossa	sapphirina	Panama	9,15210	-79,84648	278	x	x
Euglossa	sapphirina	Panama	9,15210	-79,84648	291	x	x
Euglossa	sapphirina	Panama	9,15210	-79,84648	302		x
Euglossa	sapphirina	Panama	9,15210	-79,84648	304		x
Euglossa	sapphirina	Costa Rica	9,62626	-82,84993	1673	x	x
Euglossa	sapphirina	Costa Rica	8,70342	-83,18755	1624		x
Euglossa	sapphirina	Costa Rica	8,70342	-83,18755	1625		x
Euglossa	sapphirina	Costa Rica	8,70342	-83,18755	1633		x
Euglossa	sapphirina	Costa Rica	8,70342	-83,18755	1649		x
Euglossa	sapphirina	Costa Rica	8,70342	-83,18755	1650		x
Euglossa	sapphirina	Costa Rica	8,70342	-83,18755	1651		x
Euglossa	sapphirina	Costa Rica	8,70342	-83,18755	1654		x
Euglossa	sapphirina	Colombia	5,58895	-75,86719	CAH-046		x
Euglossa	sapphirina	Panama	8,85991	-79,87868	350	x	
Euglossa	sapphirina	Costa Rica	10,39896	-84,13506	432	x	x
Euglossa	sapphirina	Costa Rica	10,39896	-84,13506	433	x	x
Euglossa	sapphirina	Costa Rica	10,39896	-84,13506	434	x	x
Euglossa	stilbonota	Colombia	-4,20543	-69,93281	CAH-059	x	
Euglossa	stilbonota	Colombia	-4,20543	-69,93281	CAH-100	x	
Euglossa	stilbonota	Colombia	-4,20543	-69,93281	CAH-101	x	
Euglossa	stilbonota	Colombia	-4,20543	-69,93281	CAH-102		x
Euglossa	stilbonota	Colombia	-4,20543	-69,93281	CAH-104	x	x
Euglossa	stilbonota	Colombia	-4,20543	-69,93281	CAH-105	x	x
Euglossa	stilbonota	Colombia	-4,20543	-69,93281	CAH-109	x	x
Euglossa	stilbonota	Guyane	4,54893	-52,49186	1319	x	
Euglossa	stilbonota	Guyane	4,78401	-52,43075	1234	x	
Euglossa	stilbonota	Guyane	4,78401	-52,43075	1237	x	
Euglossa	stilbonota	Guyane	4,78401	-52,43075	1241	x	
Euglossa	stilbonota	Guyane	4,78401	-52,43075	1261	x	
Euglossa	stilbonota	Guyane	4,78401	-52,43075	1577	x	
Euglossa	stilbonota	Guyane	4,53861	-52,13694	1272	x	
Euglossa	stilbonota	Guyane	4,53861	-52,13694	1277		x
Euglossa	stilbonota	Guyane	4,53861	-52,13694	1283	x	
Euglossa	tridentata	Panama	9,15210	-79,84648	298		x
Euglossa	tridentata	Panama	9,15210	-79,84648	301	x	x

Euglossa	tridentata	Colombia	6,08334	-77,41670	CAH-162		x
Euglossa	tridentata	Colombia	6,08334	-77,41670	CAH-170		x
Euglossa	tridentata	Colombia	6,08334	-77,41670	CAH-228	x	x
Euglossa	tridentata	Colombia	6,08334	-77,41670	CAH-233	x	x
Euglossa	tridentata	Costa Rica	8,70342	-83,18755	488	x	
Euglossa	tridentata	Mexico	17,07920	-97,86740	652	x	
Euglossa	tridentata	Mexico	17,07920	-97,86740	700	x	
Euglossa	tridentata	Mexico	16,60344	-90,91443	25	x	
Euglossa	tridentata	Mexico	16,60344	-90,91443	27	x	
Euglossa	tridentata	Mexico	16,60344	-90,91443	28	x	
Euglossa	tridentata	Mexico	18,64347	-95,09647	3	x	
Euglossa	tridentata	Mexico	17,51020	-91,98104	11	x	
Euglossa	tridentata	Mexico	17,51020	-91,98104	12	x	
Euglossa	tridentata	Mexico	17,51020	-91,98104	13	x	
Euglossa	tridentata	Mexico	20,62009	-97,68576	602	x	x
Euglossa	tridentata	Mexico	20,62009	-97,68576	614		x
Euglossa	tridentata	Colombia	5,58895	-75,86719	CAH-004		x
Euglossa	tridentata	Costa Rica	10,39896	-84,13506	437	x	
Euglossa	turbinifex	Colombia	-4,20543	-69,93281	CAH-108		x
Euglossa	turbinifex	Colombia	6,08334	-77,41670	CAH-183	x	x
Euglossa	turbinifex	Colombia	6,08334	-77,41670	CAH-196	x	x
Euglossa	turbinifex	Colombia	6,08334	-77,41670	CAH-197	x	x
Euglossa	turbinifex	Colombia	6,08334	-77,41670	CAH-198	x	x
Euglossa	turbinifex	Colombia	6,08334	-77,41670	CAH-235	x	x
Euglossa	variabilis	Mexico	21,42595	-87,34012	371		x
Euglossa	variabilis	Mexico	20,40037	-97,33705	1	x	
Euglossa	variabilis	Colombia	6,08334	-77,41670	CAH-230	x	x
Euglossa	variabilis	Mexico	17,07920	-97,86740	667	x	
Euglossa	variabilis	Mexico	18,99228	-96,13856	115	x	x
Euglossa	variabilis	Mexico	18,99228	-96,13856	116	x	x
Euglossa	variabilis	Mexico	18,99228	-96,13856	118	x	x
Euglossa	variabilis	Panama	9,55390	-79,65580	1186	x	
Euglossa	variabilis	Mexico	20,62009	-97,68576	603	x	x
Euglossa	variabilis	Mexico	20,62009	-97,68576	616	x	
Euglossa	variabilis	Mexico	20,62009	-97,68576	622	x	x
Euglossa	variabilis	Mexico	20,62009	-97,68576	631	x	x
Euglossa	variabilis	Mexico	20,62009	-97,68576	633	x	
Euglossa	variabilis	Mexico	20,62009	-97,68576	752	x	
Euglossa	variabilis	Mexico	20,62009	-97,68576	757	x	
Euglossa	variabilis	Mexico	20,62009	-97,68576	822	x	
Euglossa	variabilis	Colombia	5,58895	-75,86719	CAH-003		x
Euglossa	variabilis	Colombia	5,58895	-75,86719	CAH-026		x
Euglossa	villosa	Mexico	17,07920	-97,86740	659	x	x
Euglossa	villosa	Mexico	17,07920	-97,86740	692	x	x
Euglossa	villosa	Mexico	18,99228	-96,13856	1046	x	x
Euglossa	villosa	Mexico	20,62009	-97,68576	756	x	x
Euglossa	villosa	Mexico	20,62009	-97,68576	812	x	

Euglossa	villosa	Mexico	20,62009	-97,68576	1105	x	x
Euglossa	villosa	Mexico	17,38921	-97,85516	729		x
Euglossa	villosa	Mexico	17,38921	-97,85516	749	x	x
Euglossa	villosa	Mexico	17,04520	-96,75491	163	x	
Euglossa	villosiventris	Panama	9,15210	-79,84648	293	x	
Euglossa	villosiventris	Panama	9,15210	-79,84648	309	x	
Euglossa	villosiventris	Panama	9,15210	-79,84648	311	x	
Euglossa	villosiventris	Panama	9,15210	-79,84648	313	x	x
Euglossa	villosiventris	Costa Rica	8,70342	-83,18755	462	x	x
Euglossa	villosiventris	Costa Rica	8,70342	-83,18755	463	x	x
Euglossa	villosiventris	Costa Rica	8,70342	-83,18755	472	x	
Euglossa	villosiventris	Costa Rica	8,70342	-83,18755	473	x	
Euglossa	villosiventris	Costa Rica	8,70342	-83,18755	487	x	
Euglossa	villosiventris	Costa Rica	8,70342	-83,18755	1602	x	x
Euglossa	villosiventris	Costa Rica	8,70342	-83,18755	1604	x	x
Euglossa	villosiventris	Costa Rica	8,70342	-83,18755	1648		x
Euglossa	villosiventris	Costa Rica	8,70342	-83,18755	1652	x	x
Euglossa	villosiventris	Costa Rica	8,70342	-83,18755	1653	x	x
Euglossa	viridis	Colombia	-4,20543	-69,93281	CAH-110	x	x
Euglossa	viridis	Colombia	-4,20543	-69,93281	CAH-111	x	x
Euglossa	viridis	Guyane	4,78401	-52,43075	1244	x	
Euglossa	viridis	Guyane	4,78401	-52,43075	1428	x	x
Euglossa	viridis	Guyane	4,78401	-52,43075	1433	x	
Euglossa	viridis	Guyane	4,30968	-52,13418	1488	x	
Euglossa	viridis	Guyane	4,30968	-52,13418	1493	x	x
Euglossa	viridis	Guyane	5,09747	-52,69425	1465	x	x
Euglossa	viridis	Guyane	5,09747	-52,69425	1501	x	x
Euglossa	viridis	Guyane	5,09747	-52,69425	1504	x	x
Euglossa	viridissima	Mexico	18,58769	-92,23355	392	x	
Euglossa	viridissima	Mexico	21,42595	-87,34012	375		x
Euglossa	viridissima	Mexico	21,42595	-87,34012	376		x
Euglossa	viridissima	Mexico	21,42595	-87,34012	377	x	x
Euglossa	viridissima	Mexico	16,60344	-90,91443	19	x	
Euglossa	viridissima	Mexico	18,99228	-96,13856	119	x	
Euglossa	viridissima	Mexico	18,99228	-96,13856	120	x	
Euglossa	viridissima	Mexico	18,99228	-96,13856	122	x	
Euglossa	viridissima	Mexico	18,99228	-96,13856	124	x	
Euglossa	viridissima	Mexico	18,99228	-96,13856	126	x	
Euglossa	viridissima	Mexico	18,99228	-96,13856	127	x	
Euglossa	viridissima	Mexico	17,51020	-91,98104	48		x
Euglossa	viridissima	Mexico	17,51020	-91,98104	49		x
Euglossa	viridissima	Mexico	17,51020	-91,98104	50		x
Euglossa	viridissima	Mexico	17,51020	-91,98104	58		x
Euglossa	viridissima	Mexico	17,51020	-91,98104	59		x
Euglossa	viridissima	Mexico	17,51020	-91,98104	60		x
Euglossa	viridissima	Mexico	17,51020	-91,98104	61		x
Euglossa	viridissima	Mexico	17,51020	-91,98104	62	x	x

Euglossa	viridissima	Mexico	20,62009	-97,68576	638	x	
Euglossa	viridissima	Mexico	20,62009	-97,68576	772	x	x
Euglossa	tridentata	Mexico	18,99228	-96,13856	104	x	x
Euglossa	tridentata	Mexico	18,99228	-96,13856	105	x	
Euglossa	tridentata	Mexico	18,99228	-96,13856	106	x	
Euglossa	variabilis	Mexico	18,99228	-96,13856	108	x	x
Eulaema	bombiformis	Colombia	-4,20543	-69,93281	CAH-048	x	x
Eulaema	bombiformis	Colombia	-4,20543	-69,93281	CAH-049	x	x
Eulaema	bombiformis	Colombia	-4,20543	-69,93281	CAH-061	x	x
Eulaema	bombiformis	Colombia	-4,20543	-69,93281	CAH-136	x	x
Eulaema	bombiformis	Colombia	-4,20543	-69,93281	CAH-138	x	x
Eulaema	bombiformis	Guyane	4,78401	-52,43075	1265	x	
Eulaema	bombiformis	Guyane	4,78401	-52,43075	1266	x	
Eulaema	cingulata	Colombia	-4,20543	-69,93281	CAH-050	x	x
Eulaema	cingulata	Colombia	-4,20543	-69,93281	CAH-094	x	x
Eulaema	cingulata	Colombia	-4,20543	-69,93281	CAH-137	x	x
Eulaema	cingulata	Panama	9,15210	-79,84648	308	x	x
Eulaema	cingulata	Guyane	4,54893	-52,49186	1328	x	x
Eulaema	cingulata	Guyane	4,54893	-52,49186	1330	x	x
Eulaema	cingulata	Guyane	4,54893	-52,49186	1331	x	x
Eulaema	cingulata	Guyane	4,78401	-52,43075	1264	x	
Eulaema	cingulata	Guyane	4,78401	-52,43075	1268	x	
Eulaema	cingulata	Guyane	4,78401	-52,43075	1269	x	
Eulaema	cingulata	Mexico	17,04520	-96,75491	219	x	x
Eulaema	meriana	Colombia	-4,20543	-69,93281	CAH-093	x	x
Eulaema	meriana	Panama	9,15210	-79,84648	286	x	x
Eulaema	meriana	Panama	9,15210	-79,84648	288	x	x
Eulaema	meriana	Panama	9,15210	-79,84648	289	x	x
Eulaema	meriana	Colombia	6,08334	-77,41670	CAH-156	x	x
Eulaema	meriana	Colombia	6,08334	-77,41670	CAH-157	x	x
Eulaema	meriana	Colombia	6,08334	-77,41670	CAH-188	x	x
Eulaema	meriana	Colombia	6,08334	-77,41670	CAH-189	x	x
Eulaema	meriana	Colombia	6,08334	-77,41670	CAH-190	x	x
Eulaema	meriana	Guyane	4,78401	-52,43075	1267	x	
Eulaema	meriana	Guyane	4,53861	-52,13694	1293	x	x
Eulaema	meriana	Colombia	5,58895	-75,86719	CAH-024	x	x
Eulaema	meriana	Colombia	5,58895	-75,86719	CAH-032	x	x
Eulaema	meriana	Guyane	5,15626	-52,88575	1393	x	x
Eulaema	nigrita	Panama	9,15210	-79,84648	315	x	x
Eulaema	nigrita	Panama	9,15210	-79,84648	316	x	x
Eulaema	nigrita	Guyane	4,78401	-52,43075	1434	x	x
Eulaema	nigrita	Guyane	5,09747	-52,69425	1436	x	x
Eulaema	polychroma	Mexico	20,86847	-89,62453	45	x	x
Eulaema	polychroma	Mexico	20,86847	-89,62453	46	x	x
Eulaema	polychroma	Mexico	20,86847	-89,62453	47	x	x
Exaerete	frontalis	Colombia	-4,20543	-69,93281	CAH-113	x	x
Exaerete	frontalis	Colombia	-4,20543	-69,93281	CAH-114	x	x

Exaerete	frontalis	Panama	9,15210	-79,84648	290	x	x
Exaerete	frontalis	Panama	9,15210	-79,84648	307	x	x
Exaerete	frontalis	Panama	9,15210	-79,84648	317	x	x
Exaerete	frontalis	Panama	9,15210	-79,84648	318	x	x
Exaerete	frontalis	Panama	9,15210	-79,84648	319	x	x
Exaerete	frontalis	Panama	9,15210	-79,84648	320	x	x
Exaerete	frontalis	Guyane	4,54893	-52,49186	1326		x
Exaerete	frontalis	Guyane	4,54893	-52,49186	1327		x
Exaerete	frontalis	Colombia	6,08334	-77,41670	CAH-191	x	x
Exaerete	frontalis	Colombia	6,08334	-77,41670	CAH-192	x	x
Exaerete	frontalis	Mexico	16,60344	-90,91443	37	x	
Exaerete	frontalis	Mexico	16,60344	-90,91443	38	x	
Exaerete	frontalis	Mexico	16,60344	-90,91443	39	x	
Exaerete	frontalis	Guyane	4,53861	-52,13694	1290	x	x
Exaerete	frontalis	Guyane	4,83111	-52,44389	1345	x	x
Exaerete	frontalis	Guyane	4,83111	-52,44389	1346	x	x
Exaerete	frontalis	Mexico	17,04520	-96,75491	213	x	x
Exaerete	frontalis	Mexico	17,04520	-96,75491	214	x	x
Exaerete	frontalis	Mexico	17,04520	-96,75491	215	x	
Exaerete	frontalis	Mexico	17,04520	-96,75491	216	x	
Exaerete	frontalis	Mexico	17,04520	-96,75491	217	x	
Exaerete	frontalis	Colombia	5,58895	-75,86719	CAH-001	x	x
Exaerete	frontalis	Colombia	5,58895	-75,86719	CAH-025	x	x
Exaerete	frontalis	Guyane	5,15626	-52,88575	1392	x	x
Exaerete	smaragdina	Ecuador	-0,79913	-75,51029	CAH-262	x	x
Exaerete	smaragdina	Ecuador	-0,79913	-75,51029	CAH-263	x	x
Exaerete	smaragdina	Ecuador	-0,79913	-75,51029	CAH-264	x	x
Exaerete	smaragdina	Ecuador	-0,79913	-75,51029	CAH-313	x	x
Exaerete	smaragdina	Colombia	-4,20543	-69,93281	CAH-115	x	x
Exaerete	smaragdina	Guyane	4,78401	-52,43075	1360	x	x
Exaerete	smaragdina	Guyane	4,53861	-52,13694	1289	x	x
Exaerete	smaragdina	Guyane	5,09747	-52,69425	1508	x	x
Exaerete	smaragdina	Guyane	5,09747	-52,69425	1509	x	x
Exaerete	smaragdina	Mexico	17,51020	-91,98104	4	x	
Exaerete	smaragdina	Mexico	17,51020	-91,98104	5	x	
Exaerete	smaragdina	Mexico	17,51020	-91,98104	6	x	
Exaerete	smaragdina	Mexico	17,04520	-96,75491	218	x	x
Exearete	smaragdina	Mexico	17,07920	-97,86740	925	x	x
Exearete	smaragdina	Mexico	18,99228	-96,13856	893	x	x
Exearete	smaragdina	Mexico	20,62009	-97,68576	763	x	x
Exearete	smaragdina	Mexico	17,38921	-97,85516	748	x	
Exearete	smaragdina	Mexico	17,38921	-97,85516	750	x	x
