# Supplementary material for: Evolution of Acquired Perfumes and Endogenous Lipid Secretions in Orchid Bees
Source: J Chem Ecol. 2024 Jul 3;50(9-10):430–8. doi: 10.1007/s10886-024-01514-w (PMC11493807; doi:10.1007/s10886-024-01514-w)
Supplement: Supplementary file 4 — Supplementary file4 Supplementary Information 4 (DOCX 1.03 MB) [file 10886_2024_1514_MOESM4_ESM.docx]

Fig. 1 Two-dimensional chemospaces of male hind tibial perfumes (**A**) and cephalic labial gland secretions (**B**) in relation to the phylogeny of orchid bees (Euglossini). Distances between individual dots (species) represent dissimilarity (Bray–Curtis) of chemical profiles among species reduced in dimensionality with n-MDS. Connecting lines reflect unrooted phylogenetic relationships taken from Ramirez et al. (2010a). Euglossine genera are color coded: *Exaerete* (red), *Eufriesea* (green), *Aglae* (brown), *Eulaema* (blue) and *Euglossa* (turquois)
